# Inhibition of CFTR-mediated intestinal chloride secretion by nornidulin: Cellular mechanisms and anti-secretory efficacy in human intestinal epithelial cells and human colonoids

**DOI:** 10.1371/journal.pone.0314723

**Published:** 2024-12-23

**Authors:** Chamnan Yibcharoenporn, Thidarat Kongkaew, Nichakorn Worakajit, Rungtiwa Khumjiang, Praphatsorn Saetang, Saravut Satitsri, Vatcharin Rukachaisirikul, Chatchai Muanprasat

**Affiliations:** 1 Chakri Naruebodindra Medical Institute, Faculty of Medicine Ramathibodi Hospital, Mahidol University, Bang Phli, Samut Prakarn, Thailand; 2 Translational Medicine, Faculty of Medicine Ramathibodi Hospital, Mahidol University, Bangkok, Thailand; 3 Department of Science, Faculty of Science and Technology, Prince of Songkla University, Pattani, Thailand; 4 Division of Physical Science and Center of Excellence for Innovation in Chemistry, Faculty of Science, Prince of Songkla University, Hat Yai, Songkhla, Thailand; University of Illinois at Chicago, UNITED STATES OF AMERICA

## Abstract

Secretory diarrhea, a major global health concern, particularly among young children, is often characterized by excessive chloride secretion through the cystic fibrosis transmembrane conductance regulator (CFTR) channel. Nornidulin, a fungus-derived natural product from *Aspergillus unguis*, has previously been shown to inhibit cAMP-induced Cl^-^ secretion in T84 cells (human intestinal cell lines). However, the cellular mechanism of nornidulin in inhibiting cAMP-induced Cl^-^ secretion and its anti-secretory efficacy is still unknown especially in a human colonoid model, a preclinical model recapitulating intestinal physiology in humans. This research study aimed to examine the mechanism of nornidulin to inhibit cAMP-induced chloride secretion and assess its ability to reduce fluid secretion in both T84 cells and human colonoid models. Apical Cl^-^ current analyses showed that nornidulin inhibited CFTR-mediated Cl^-^ current in T84 cells with IC_50_ of ~1.5 μM. Nornidulin treatment had no effect on CFTR protein expression. Additionally, the inhibitory effects of nornidulin on CFTR-mediated chloride currents were unaffected by the presence of compounds that inhibit negative regulators of CFTR function, such as protein phosphatases, AMP-activated protein kinases, and phosphodiesterases. Interestingly, nornidulin suppressed the increase in intracellular cAMP levels caused by forskolin, an activator of adenylate cyclases, in T84 cells. Using human colonoid models, we found that nornidulin significantly suppressed the forskolin and cholera toxin-induced fluid secretion, indicating that nornidulin exerted an anti-secretory effect in human intestinal epithelia. Collectively, nornidulin represents a novel class of fungus-derived inhibitors of CFTR-mediated Cl^-^ secretion, potentially making it a promising candidate for the development of anti-secretory treatments.

## Introduction

Secretory diarrhea remains an important health problem, especially in developing countries. This disease is more complex and severe in children under five years old, which accounts for ~1–2 billion cases per year worldwide [1]. The mortality rate of secretory diarrheas in children under five years of age is highest in five countries, including Nigeria, China, India, Pakistan, and Congo, despite the use of fluid replacement as mainstay therapy [2]. Therefore, there is a need to develop a novel adjunctive therapy to reduce the severity of secretory diarrheas, particularly by targeting their pathogenic mechanisms. The pathophysiology of secretory diarrheas involves excessive intestinal fluid and Cl^-^ secretion, triggered by various etiological agents and compounds including bacterial enterotoxins (e.g. cholera toxin), bile acids, and medications (e.g. flavopiridol and afatinib) [3, 4]. Indeed, the process of intestinal Cl^-^ secretion is facilitated by two primary mechanisms: Cl^-^ uptake via Na^+^-K^+^-2Cl^-^ cotransporters and Cl^-^ efflux through apical Cl^-^ channels, predominantly the cystic fibrosis transmembrane conductance regulator (CFTR). Furthermore, Na^+^-K^+^ ATPases and K^+^ channels located on the basolateral membrane play a crucial role in maintaining the electrochemical gradient necessary for sustained Cl^-^ secretion [5, 6].

CFTR, a cAMP-activated chloride channel, is a member of the ATP-binding cassette superfamily. It is composed of five primary domains: two transmembrane domains (TMD1 and TMD2), which are connected to two nucleotide-binding domains (NBD1 and NBD2) at the cytosolic sites. The NBDs are also connected to the regulatory domain (R domain) [7]. TMDs consist of 12 membrane-spanning domains, which are equally separated into TMD1 and 2. TMD1 and TMD2 are connected to form an anion gate at the plasma membrane, allowing transport of anions especially Cl^-^. NBDs at the cytosolic sites, where ATP is bound and hydrolyzed, enabling dimerization of NBDs and opening of the gate [8]. Furthermore, the R domain, located between the NBDs, undergoes phosphorylation by cAMP-dependent protein kinase A. This phosphorylation enhances both the open probability and ATP hydrolysis of CFTR [9, 10]. Negative regulators of CFTR activities include phosphodiesterases (PDE), which degrade cAMP, protein phosphatases (PP) especially type 2A and 2C, which dephosphorylate CFTR, and AMP-activated protein kinases (AMPK), which phosphorylate at the inhibitory sites of R domains [10, 11].

Up to now, researchers have identified several CFTR inhibitors that have demonstrated efficacy in treating secretory diarrheas in animal models of enterotoxin-induced intestinal disorders [12–14]. CFTR inhibitors are categorized into direct and indirect CFTR inhibitors based on their mechanism of action. Direct CFTR inhibitors exert their inhibitory effect directly on the CFTR e.g. CFTR_inh_-172 and GlyH-101 [15]. Indirect CFTR inhibitors exert their effect on other proteins regulating CFTR functions e.g. CHAL-025 and flufenamic acid, which have been shown to inhibit CFTR by mechanisms involving AMPK activation [16, 17]. Natural resources including plants and microorganisms are regarded as important sources of bioactive compounds for drug discovery [18]. Indeed, several types of compounds from fungi including arthropsolide A, zearalenone, and α,β-dehydrolovastatin have demonstrated inhibitory effects on CFTR-mediated intestinal Cl^-^ secretion and shown efficacy in reducing enterotoxin-induced fluid secretion in murine models [19–21]. Interestingly, nornidulin, a depsidone derivative isolated from the soil fungus *Aspergillus unguis* PSU-RSPG204, has been shown to inhibit CFTR-mediated Cl^-^ secretion in human intestinal epithelial T84 cells [22]. Nevertheless, the precise mechanism of action and anti-secretory efficacy of nornidulin in human colonoid models, which serve as *ex vivo* representations of the human colon, remain unelucidated. This investigation sought to elucidate the cellular mechanisms underlying CFTR inhibition by nornidulin and to assess its potential as an anti-secretory agent using both T84 cells and human colonoid models [23].

## Material and methods

### Chemical reagents

Nornidulin was isolated from both the soil-derived fungus *Aspergillus unguis* PSU-RSPG204 and the marine-derived *A*. *unguis* PSU-MF16, as previously described [22, 24]. Purity of nornidulin (>98%) was analyzed by TLC chromatogram and the ^1^H NMR spectrum. The NMR spectroscopic data and the NMR spectra of nornidulin were shown in S1 File. Nornidulin was dissolved in DMSO. Fetal bovine serum (FBS), culture media, trypsin, penicillin, and streptomycin were purchased from Thermo Fisher Scientific Inc. (Waltham, MA, USA). Antibodies against CFTR and β-actin were purchased from Cell Signaling Technology (Boston, MA, USA). Cholera toxins from *Vibrio cholerae* (product no.100B: Lot no. 10071A2) were purchased from List Biological Laboratories (Campbell, CA, USA). Other compounds and chemicals were purchased from Sigma-Aldrich (St. Louis, MO, USA).

### Cell culture

T84 cells at passage 10–30 from the American Type Culture Collection (Manassas, VA, USA) were cultured in a mixture of Dulbecco’s Modified Eagle’s medium and Ham’s F-12 medium (1:1) supplemented with 5% FBS, 100 U/mL penicillin, and 100 μg/mL streptomycin. T84 cells were cultured in a humidified incubator under an atmosphere of 95% O_2_/ 5% CO_2_ at 37°C. The culture medium was changed every two days, and cells were split at 80% confluence.

### Measurement of short-circuit current (I_sc_) and apical Cl^-^ current (I_Cl_^-^)

T84 cells were seeded on the Snapwell polycarbonate inserts with 0.4 μm pore polyester membrane at a density of 5×10^5^ cells/insert (Costar, New York, NY, U.S.A.). The cultured media were replaced daily for two weeks, when the transepithelial electrical resistance of T84 monolayers measured by Millicell ERS-2 volt-ohm meter (Merck Ltd., An affiliate of Merck KGaA, Darmstadt, Germany) was >1,000 Ω.cm^2^. For *I*_sc_ measurements, the insert was mounted into the Ussing chambers, in which both hemichambers were filled with symmetrical Kreb’s solutions (pH 7.4) containing 120 mM NaCl, 25 mM NaHCO_3_, 3.3 mM KH_2_PO_4_, 0.8 mM K_2_HPO_4_, 1.2 mM MgCl_2_, 1.2 mM CaCl_2_ and 10 mM glucose. For apical *I*_Cl_^-^ measurements, solutions with low Cl^-^ and high Cl^-^ concentrations (pH 7.4) were added into apical and basolateral hemichambers, respectively, to generate the basolateral-to-apical Cl^-^ gradient. The high Cl^-^ solutions contained 130 mM NaCl, 2.7 mM KCl, 1.5 mM KH_2_PO_4_, 1 mM CaCl_2_, 0.5 mM MgCl_2_, 10 mM Na-HEPES, and 10 mM glucose. In low Cl^-^ solutions, 65 mM NaCl was replaced by 65 mM sodium gluconate, and the concentration of CaCl_2_ was increased to 2 mM. To establish basolateral membrane permeabilization, amphotericin B was added into the basolateral hemichamber (250 μg/mL) and incubated for 30 min before apical *I*_Cl_^-^ measurement. Solutions were maintained at 37°C and bubbled with 95% O_2_/5% CO_2_ for *I*_sc_ measurement and 100% O_2_ for apical *I*_Cl_^-^ measurement, respectively. *I*_*sc*_ and apical *I*_*Cl*_^-^ were recorded by DVC-1000 voltage-clamp (World Precision Instruments, Sarasota, FL, U.S.A.) with Ag/AgCl electrodes and 3M KCl agar bridges.

### Cell viability assays

T84 cells were plated at a density of 1 × 10^5^ cells/well in 96-well plates and cultured overnight. Then, cells were cultured for 24 h with media containing DMSO (control) or nornidulin at various concentrations from 0.5 μM to 5 μM. The cells were incubated for 4 h with 3-(4,5-dimethyl-2-thiazolyl)-2,5-diphenyl-2H-tetrazolium bromide (MTT reagent) at 5 mg/mL. The blue formazan product was dissolved with DMSO. The absorbance was detected at 540 nm using a spectrophotometer.

### Western blotting analysis

T84 cells were plated onto 6-well plates at 1.5×10⁶ cells per well. After treatment with either vehicle or nornidulin for 24 h, cell lysates were prepared using RIPA buffer containing protease inhibitors. Protein concentrations were quantified using the Bradford assay. Then, equivalent amounts of protein were separated by SDS-PAGE on 10% gel and transferred to nitrocellulose membranes. The membranes were blocked with 5% milk for 60 min and incubated overnight at 4°C with primary antibodies against CFTR and β-actin. After washing, the membranes were probed with horseradish peroxidase-conjugated secondary antibodies. Chemiluminescence was used to visualize the protein bands and their intensities were analyzed using ImageJ software. Original blots were in S2 File.

### Intracellular cAMP measurements

The intracellular cAMP level was measured by the cAMP Parameter Assay Kit (Cat# KGE002B, R&D Systems, Minneapolis, Minnesota, USA). T84 cells were plated onto 24-well plates at a density of 5x10^5^ cells/well and cultured for a day. Cells were then incubated for 1 h with DMSO, forskolin (5 μM), nornidulin (5 μM) and forskolin plus nornidulin. At the time of cAMP assay, the cells were centrifuged, and supernatants were discarded. Cells were resuspended in a cell lysis buffer (diluted 1:5) to a concentration of 1 x 10^7^ cells/mL. Conjugated horseradish peroxidase (HRP)-cAMP was used to compete with intracellular cAMP for binding to anti-cAMP antibodies. The optical density (O.D.) was measured using the multi-mode microplate reader (Biotek, Life Science, Inc., USA). Intracellular cAMP level was calculated from a standard curve of samples with known cAMP levels.

### Human colonoid swelling assays

Human epithelial crypt-like cells were obtained from colon tissue biopsies. Crypt isolation was performed as previously described with minor modifications [23]. This study has been approved by the Human Research Ethics Committee, Faculty of Medicine Ramathibodi Hospital, Mahidol University (permit number MURA2024/212). Colonoids for this study were collected from healthy subjects at Faculty of Medicine, Ramathibodi Hospital during year 2020–2021 with written informed consent. In brief, human colon tissues were washed with a chelated solution containing 5.55 mM Na _2_HPO_4_, 7.9 mM KH_2_PO_4_, 95.82 mM NaCl, 1.6 mM KCl, 43.82 mM sucrose, 54.89 mM D-sorbitol, and 0.5 mM dithiothreitol. Crypt fraction was centrifuged and embedded in 30 μL of Matrigel (catalog number 356231, Corning, NY, USA) in a 24-well plate. Human colonoids were maintained in DMEM-HG-based culture medium supplemented with 50% WRN (Wnt3A, R-spondin, Noggin) conditioned medium, 50 ng/mL EGF, 10 nM Gastrin, 0.5 μM A83-01, 10 μM Y-27632, 10 μM SB202190 and 2.5 μM CHIR99021. Human colonoids were grown in a CO_2_ incubator at 37°C (5% CO_2_) with culture media being replaced every two days. After ten passages, colonoids were cultured in a 48-well plate with 15 μL of Matrigel and 300 μL complete medium for 2–3 days. Swelling of the human colonoids was monitored by random imaging every 5 min using the BioTek Cytation^™^ 5 Cell Imaging Multi-Mode Reader after treatment with forskolin (5 μM), forskolin plus nornidulin, cholera toxin (2 μg/mL), or cholera toxin plus nornidulin. Swelling of colonoids was calculated as the relative area increased from 0 min to 60/240 min and analyzed as the area under the curve (AUC) using CellProfiler 4.2.1 program.

### Statistical analysis

All results are expressed as means ± S.E.M. The statistical analyses of the difference between control and treatment groups were tested using one-way ANOVA followed by Bonferroni’s post hoc test. The *P* value < 0.05 was considered statistically significant. GraphPad Prism software was used for all data analyses. Minimal data set was in S3 File.

## Results

### Modulation of cAMP-mediated chloride efflux by nornidulin in colonic epithelial cells (T84 line)

**Fig 1:**
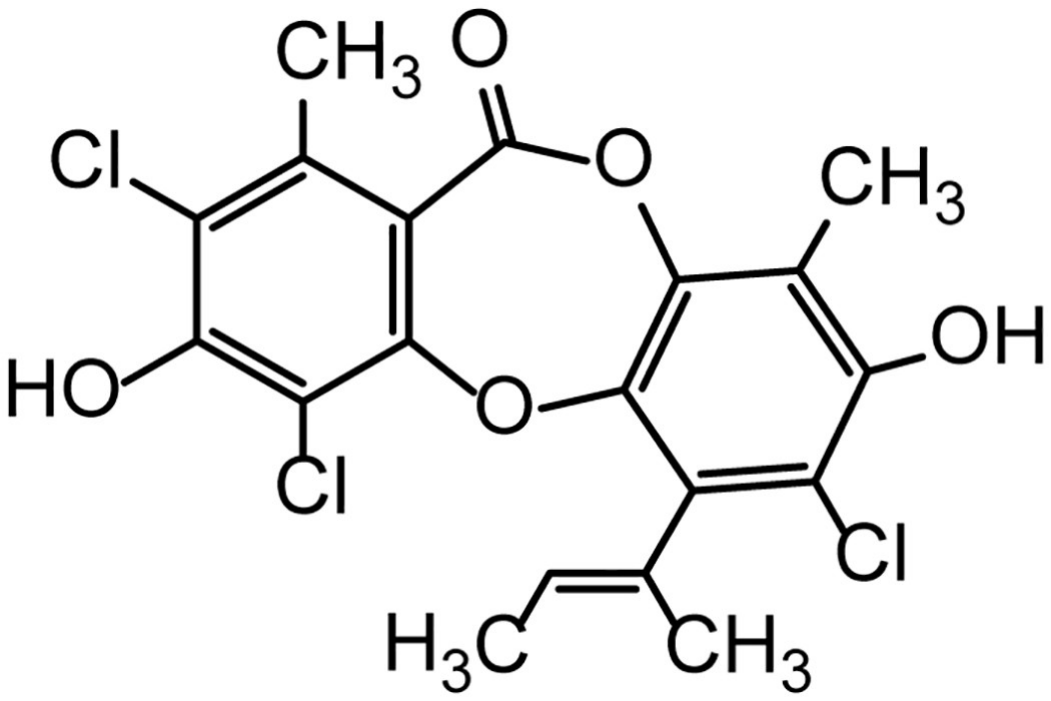
Chemical structure of nornidulin.

**Fig 2:**
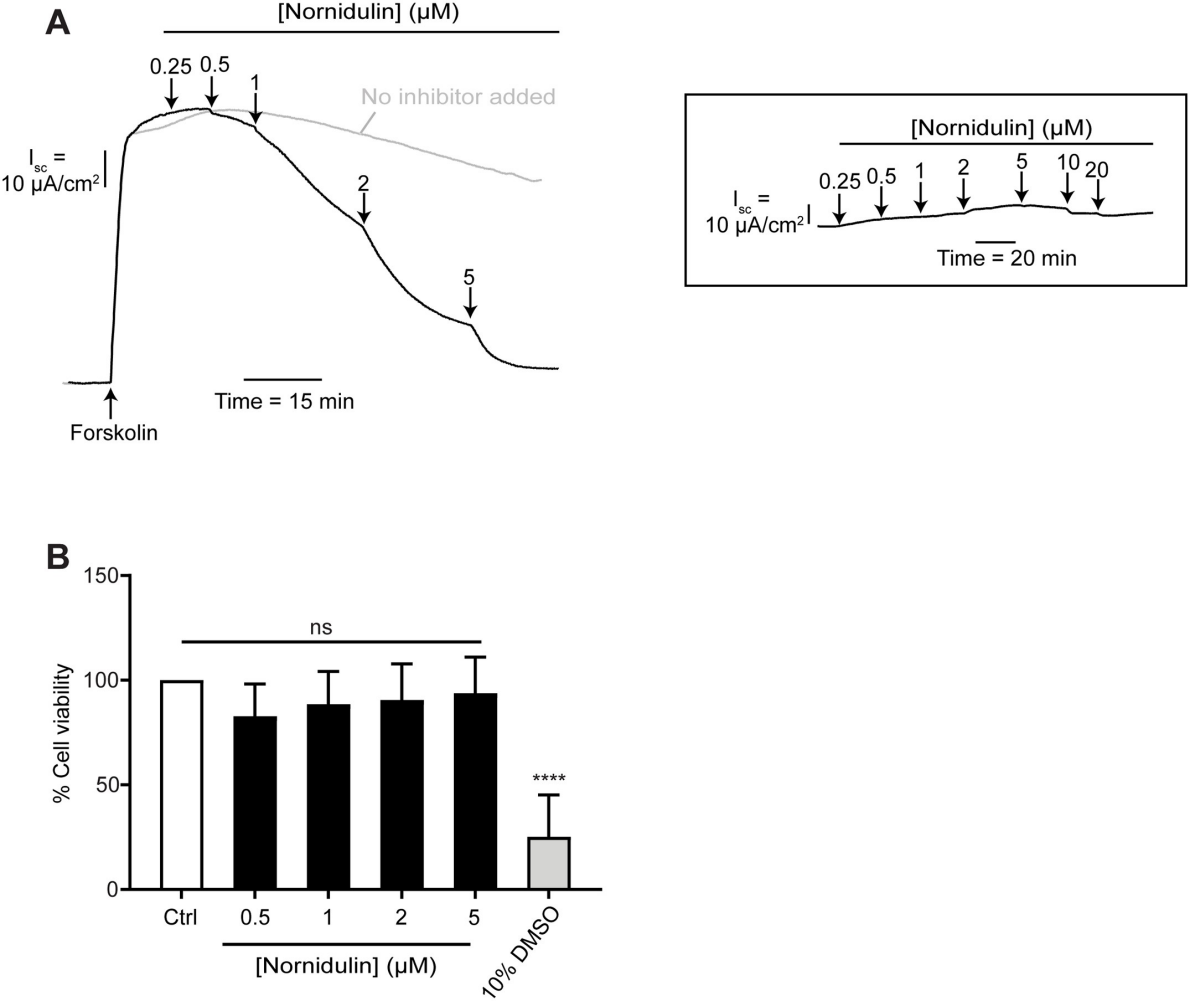
Modulation of cAMP-mediated Cl^−^ efflux and cell viability by nornudulin in T84 cells. (A) Demonstrates nornidulin’s impact on cAMP-mediated Cl^-^ efflux stimulated by 20 μM forskolin (FSK). Various concentrations of nornidulin were applied to the apical solutions (n = 5). The inset graph shows nornidulin’s effect on basal current at different concentrations (n = 5). (B) Cell viability after 24-hour exposure to varying concentrations of nornidulin in apical solutions (n = 5). A 10% DMSO solution serves as the positive control. Results are presented as percentage of cell viability relative to the control ± standard deviation (S.D.). ****, p<0.0001 compared to control, while "ns" denotes non-significant differences from the control.

Nornidulin was obtained from the extracts of culture broths of the fungi *A*. *unguis* PSU-RSPG204 and PSU-MF16 [22, 24]. The chemical structure of nornidulin was shown in Fig 1. To elucidate the inhibitory mechanism of nornidulin on cAMP-mediated chloride efflux, short-circuit current (*I*_*sc*_) was measured across T84 colonic epithelial monolayers. Forskolin (FSK), a potent adenylyl cyclase (AC) agonist, was applied apically at 20 μM to elevate intracellular cAMP levels and subsequently induce chloride secretion. Nornidulin exhibited dose-dependent inhibition of FSK-stimulated, cAMP-mediated chloride efflux, with an IC_50_ of 1.49 ± 0.14 μM and maximal inhibition at approximately 5 μM (Fig 2A). Notably, nornidulin did not alter basal *I*_*sc*_ in T84 cells (Fig 2 and inset). To assess potential cytotoxicity, T84 cells were exposed to nornidulin for 24 hours at concentrations up to 5 μM. Cell viability was quantified using the MTT assay, with 10% DMSO as a positive control for cytotoxicity. Results indicated no significant reduction in T84 cell viability at the tested nornidulin concentrations (Fig 2B).

### Effect of nornidulin on CFTR-mediated apical I_Cl_^-^ in T84 cells

**Fig 3:**
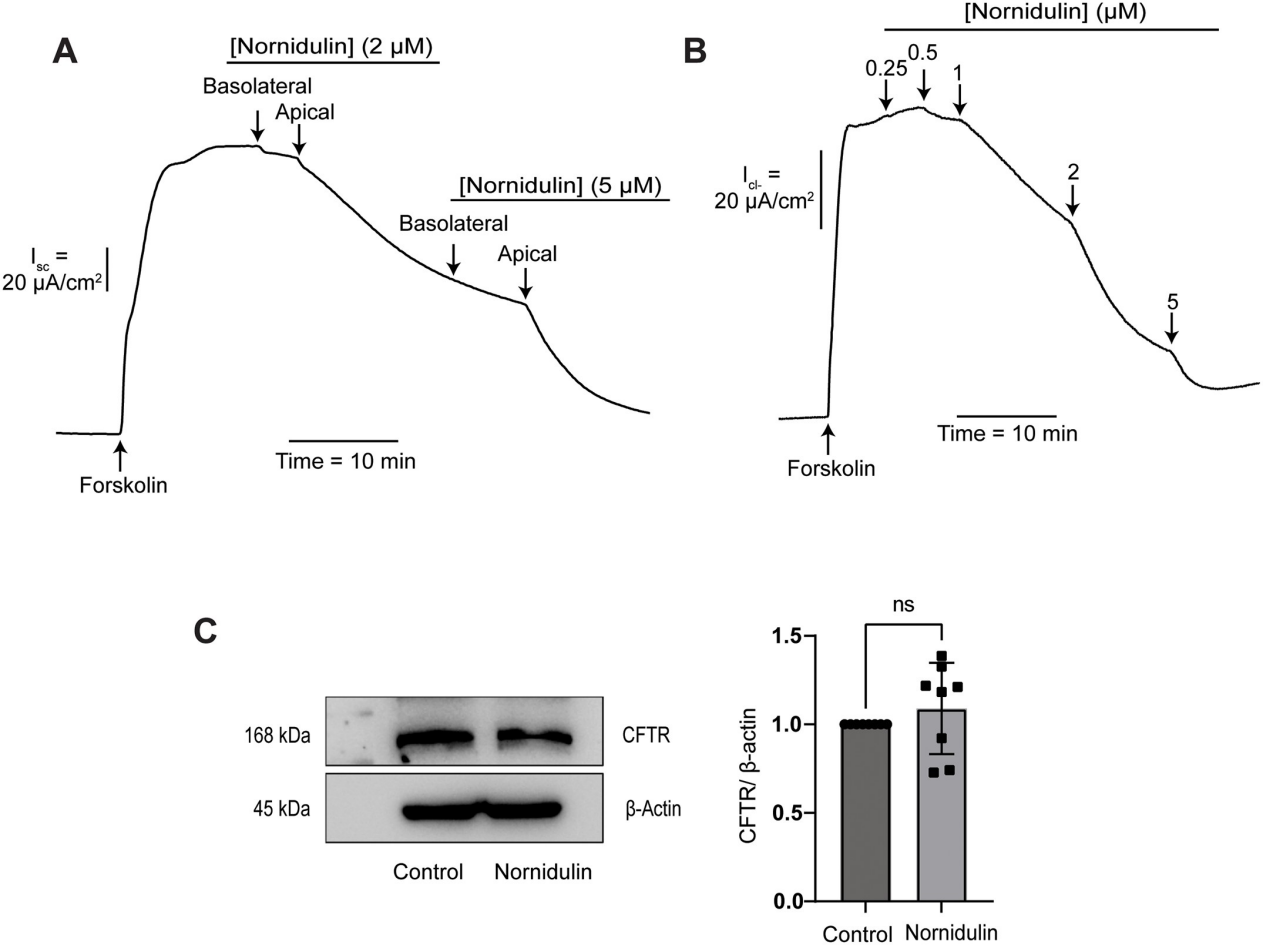
The polarity of inhibition by nornidulin on cAMP-induced Cl^−^ secretion in T84 cells. (A) Shows a representative short-circuit current (*I*_sc_) trace. In this experiment, nornidulin was applied at various concentrations to both the basolateral and apical sides of the cell layer after stimulation with FSK (n = 5). (B) Illustrates the dose-dependent response of nornidulin on CFTR-mediated apical chloride current (*I*_Cl-_) in T84 cells. Before activating CFTR with FSK, the basolateral membrane was first made permeable by using amphotericin B (250 μg/mL for 30 minutes). Nornidulin was then added to the apical side at different concentrations. The graph shows a representative trace of the apical chloride current (*I*_Cl-_) (n = 5). (C) Western blot analysis of CFTR protein expression in T84 cells after 24-h exposure to nornidulin (5 μM) (n = 8). ns, non-significant compared with control.

To determine the polarity of nornidulin’s action, *I*_*sc*_ measurements were conducted in T84 cells. Nornidulin was respectively added to both the basolateral and apical solutions at two concentrations (2 μM and 5 μM). We found that the inhibitory effect of apical additions of nornidulin on the cAMP-induced Cl^−^ secretion was higher than that of basolateral additions (Fig 3A). This result suggested that nornidulin may inhibit CFTR, a cAMP-dependent apical Cl^-^ channels in T84 cells. To confirm this hypothesis, the effect of nornidulin on CFTR-mediated Cl^-^ transport was investigated using the apical *I*_Cl_^-^ measurements in T84 cells. In this experiment, amphotericin B was used to permeabilize basolateral membrane and asymmetrical buffers of Cl^-^ ([Cl^-^] in basolateral buffers > [Cl^-^] in apical buffers) was used to generate a basolateral-to-apical Cl^-^ gradient. It was found that nornidulin inhibited the CFTR-mediated apical *I*_Cl_^-^ in a dose-dependent manner with an IC_50_ value of 1.19 ± 0.18 μM and near complete inhibition at 5 μM (Fig 3B). In addition, western blot analysis showed that 24-h incubation with nornidulin at 5 μM had no effect on CFTR protein expression in T84 cells (Fig 3C). These findings suggested that nornidulin inhibited cAMP-induced Cl^-^ secretion by reducing the activities of CFTR Cl^-^ channel.

### Mechanisms of CFTR inhibition by nornidulin in T84 cells

**Fig 4:**
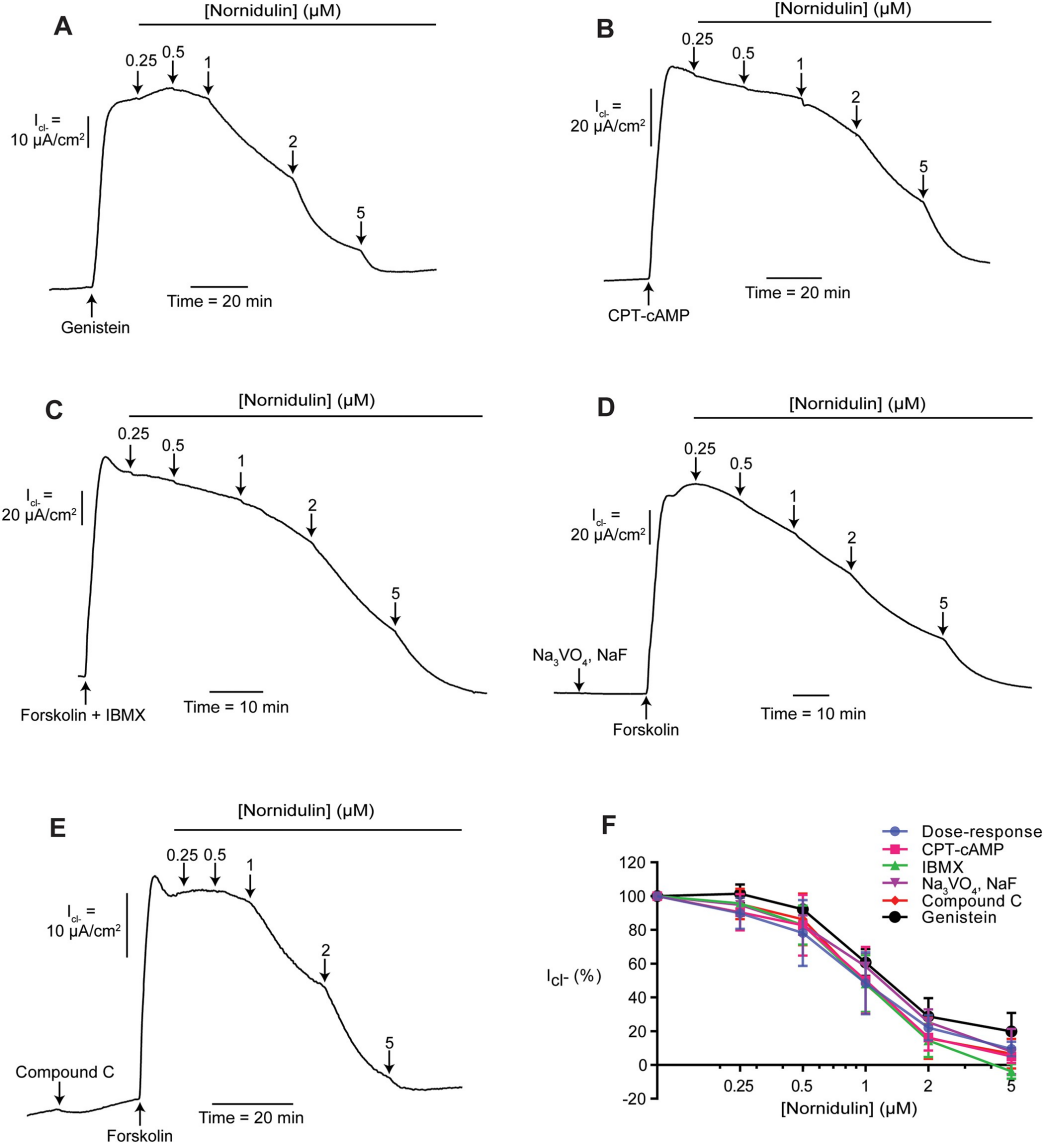
Mechanisms of CFTR inhibition by nornidulin in T84 cells. (A) A representative trace of apical chloride current (*I*_Cl-_) is shown. CFTR was first stimulated with 20 μM genistein, followed by the addition of nornidulin at various concentrations to the apical side (n = 5). (B–E) Examine how upstream regulatory proteins affect nornidulin’s inhibition of CFTR-mediated apical chloride current (*I*_Cl-_). T84 cells were pre-treated with specific activators or inhibitors: CPT-cAMP (100 μM) for PKA, IBMX (100 μM) for PDE, NaF plus Na_3_VO_4_ (1 mM) for PP, and Compound C (50 μM) for AMPK. After pre-treatment, dose-inhibition studies with nornidulin were performed, with each experiment repeated five times (n = 5). (F) Compiles dose-response curves from all the dose-inhibition studies. The data are fitted to Hill’s equation and presented as means of percentage of agonist-stimulated apical chloride current (*I*_Cl-_), with standard error of the mean (S.E.M.) indicated (n = 5).

Mechanisms of CFTR inhibition by nornidulin were investigated by both apical *I*_Cl_^-^ measurements and intracellular cAMP assays. To investigate effects of nornidulin on CFTR Cl^-^ transport activities induced by direct activation, genistein (20 μM), a direct activator of CFTR, was used to stimulate the CFTR-mediated apical *I*_Cl_^-^. We found that nornidulin inhibited the genistein-induced apical *I*_Cl_^-^ with an IC_50_ value of 1.40 ± 0.21 μM and near complete inhibition at 5 μM in T84 cells (Fig 4A and 4F). To examine whether the mechanisms of CFTR inhibition by nornidulin was downstream to cAMP and cAMP generation/degradation processes, a cell-permeable cAMP CPT-cAMP (100 μM) and FSK plus IBMX (phosphodiesterase inhibitor; 100 μM) was used as CFTR activators. Our results showed that nornidulin inhibited both CPT-cAMP and FSK plus IBMX-induced apical *I*_Cl_^-^ in T84 cells with IC_50_ values of 1.03 ± 0.13 μM and 1.13 ± 0.16 μM, respectively (Fig 4B, 4C and 4F). Furthermore, to investigate whether the inhibitory effect of nornidulin on CFTR was via protein phosphatases and AMPK, negative regulators of CFTR channel activities, T84 cells were pretreated with NaF plus Na_3_VO_4_ (protein phosphatase inhibitors; 1 mM) or compound C (AMPK inhibitor; 50 μM) before performing concentration-inhibition studies of nornidulin in T84 cells using FSK as a CFTR activator. We found that IC_50_ values of nornidulin in T84 cells pretreated with NaF plus Na_3_VO_4_ and compound C were 1.29 ± 0.12 μM and 1.09 ± 0.17 μM, respectively (Fig 4D–4F).

**Fig 5:**
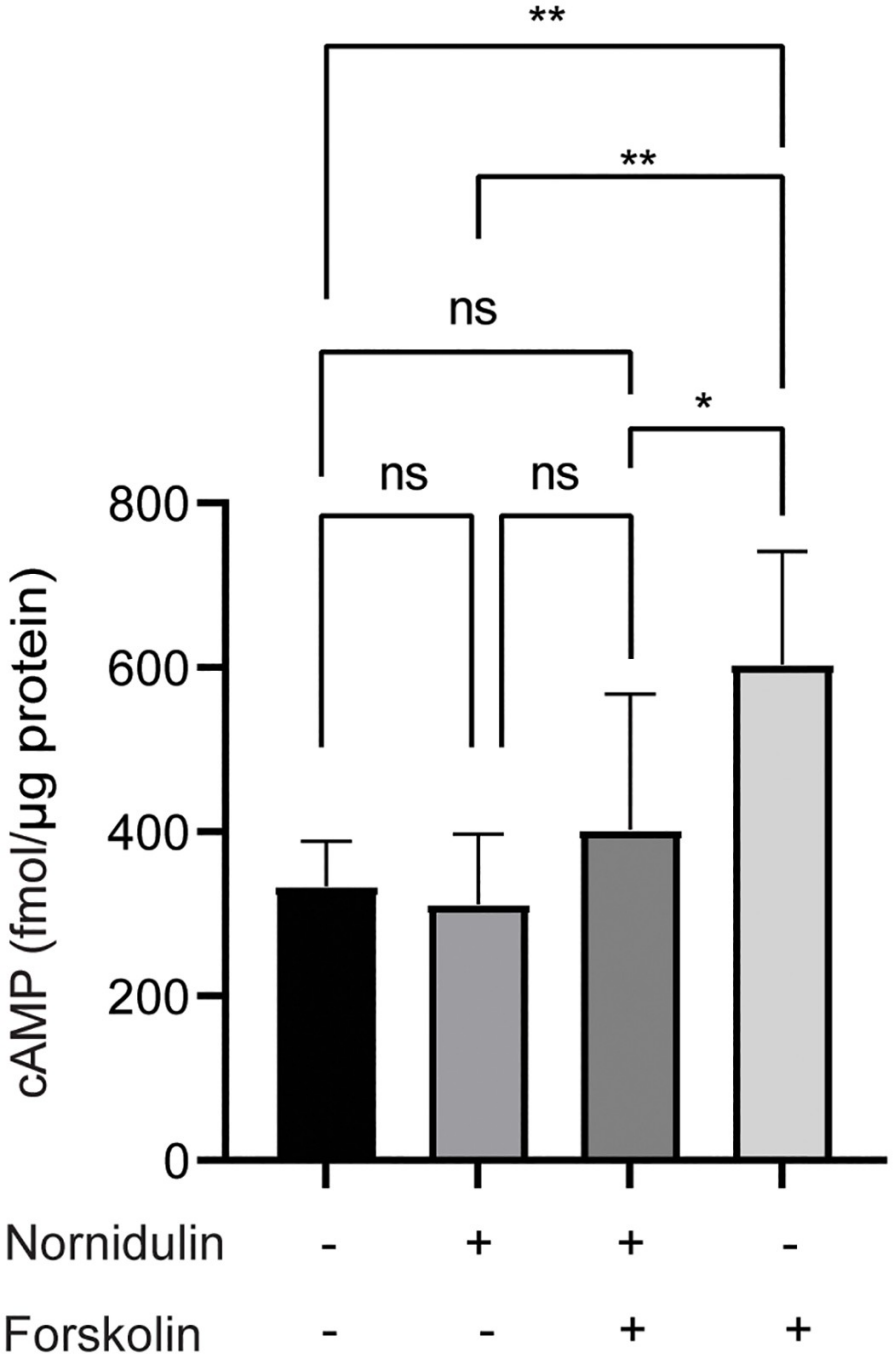
The effect of nornidulin on intracellular cAMP level. The experiment involved pretreating T84 cells for one hour with different conditions: vehicle (control), nornidulin, N9 (5 μM), nornidulin (5 μM) with FSK (5 μM), or FSK (5 μM). Following treatment, intracellular cAMP levels were determined using a cAMP assay kit. The results are expressed as cAMP concentrations with standard deviation (S.D.) (n = 5–6). ns, not significantly different from the control group. **, p <0.01 compared to the indicated group. ****, p <0.0001 compared to the indicated group.

Furthermore, effects of nornidulin on intracellular cAMP levels in T84 cells were evaluated using the cAMP Parameter Assay Kit. An-hour incubation with nornidulin showed no significant effect on basal intracellular cAMP levels compared with control (vehicle-treated group) (Fig 5). However, nornidulin significantly suppressed the FSK-induced elevation of intracellular cAMP level (Fig 5). This result indicated that nornidulin interfered with the FSK-induced cAMP elevation in T84 cells.

### Anti-secretory efficacy of nornidulin in human colonoid models

**Fig 6:**
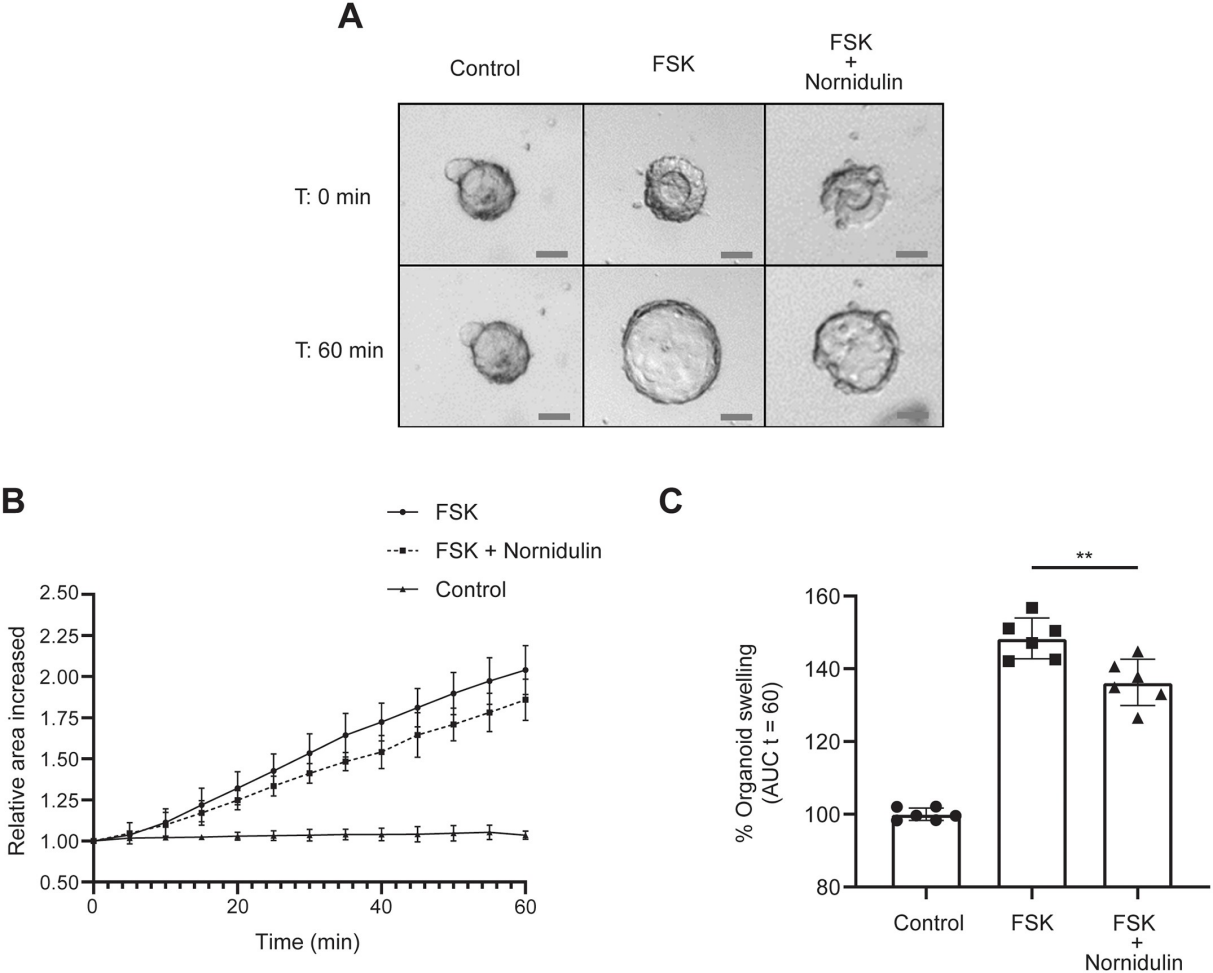
The effect of nornidulin on FSK-induced fluid secretion in human colonoid models. Effect of nornidulin on fluid secretion in human colonoids after 60 min of FSK treatment. (A) Bright-field images of colonoids after FSK (5 μM) with or without treatment with nornidulin (10 μM) at indicated time points (scale bar: 50 μm). (B) Swelling of colonoids was evaluated by relative area increased from 0 min to 60 min. (C) The area under the curve (AUC) of each condition is calculated from (B.) Each experiment was repeated independently (n = 6). **, *p* <0.01 versus FSK alone.

**Fig 7:**
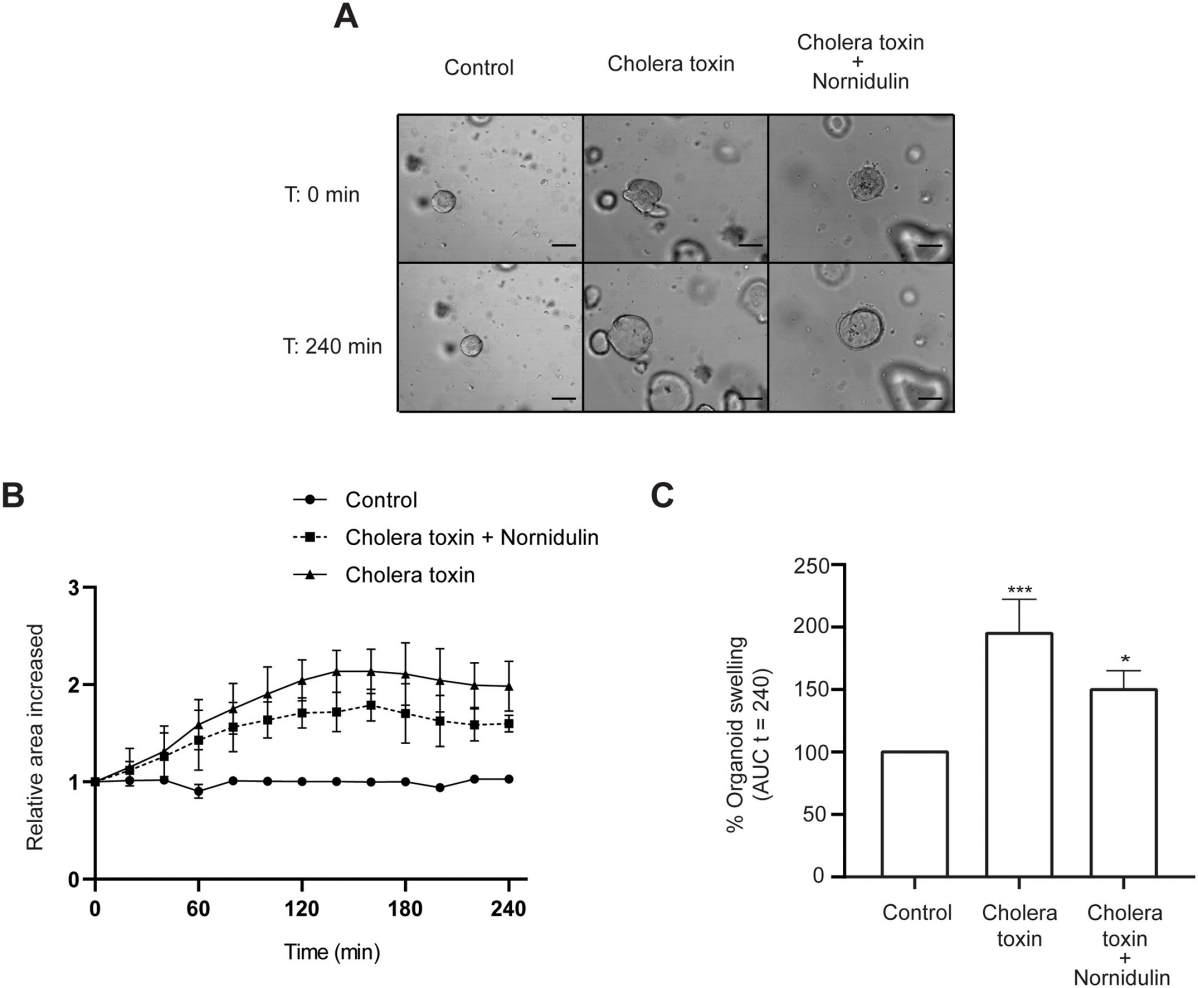
The effect of nornidulin on cholera toxin-induced fluid secretion in human colonoid models. Effect of nornidulin on fluid secretion in human colonoids after 240 min of cholera toxin treatment. (A) Bright-field images of colonoids after cholera toxin (2 μg/mL) with or without treatment with nornidulin (40 μM) at indicated time points (scale bar: 50 μm). (B) Swelling of colonoids was evaluated by relative area increased from 0 min to 240 min. (C) The area under the curve (AUC) of each condition is calculated from (B.) Each experiment was repeated independently (n = 3).***, *p* <0.001 compared to the control group. *, *p* <0.05 compared to cholera toxin-treated group.

To determine the anti-diarrheal application of nornidulin, the anti-secretory efficacy of nornidulin was evaluated using a swelling assay in human colonoid models. In this experiment, bright-field images of human colonoids were acquired at 0 and indicated time points after treatment with FSK (5 μM) or cholera toxin (CT, 2 μg/mL). After FSK treatment, the relative area of volume increased by ~ 50% within 60 min compared with control (Fig 6), reflecting the cAMP-induced intestinal fluid secretion in this model. Pre-treatment with nornidulin (10 μM) significantly suppressed the FSK-induced colonoid swelling by ~ 25%. Furthermore, CT treatment induced an increase in the relative area of volume by ~ 100% within 240 min compared with control (Fig 7). While nornidulin at 10 μM and 20 μM was found to have no significant effect on CT-induced colonoid swelling (data not shown, nornidulin at 40 μM significantly suppressed the CT-induced colonoid swelling by ~ 50% (Fig 7). These results suggested that nornidulin possessed the anti-secretory effect in the human colonoid models.

## Discussion

CFTR is an attractive target for anti-diarrheal drug discovery. Several CFTR inhibitors have been discovered from natural sources. Nornidulin, isolated from the soil-derived fungus *A*. *unguis*, is one of the compounds that exert an anti-secretory effect through CFTR Cl^-^ channels inhibition. In this study, using T84 cells, we found that nornidulin inhibited CFTR via mechanisms independent of PKA, PDE, PP, and AMPK. Importantly, nornidulin suppressed the CFTR-mediated fluid secretion in human intestinal organoids.

Nornidulin is a depsidone derivative isolated from various species of fungi. It exhibits multiple biological activities including anti-aromatase, antibacterial, antifungal, antimalarial, and antidiarrheal activities [22, 25–27]. The chemical properties of nornidulin have been depicted in previous studies [22, 28]. According to Lipinski’s rule of five, the orally administered drugs should follow five criteria: molecular weight (MW) < 500 g/mol, a calculated octanol-water partition coefficient (Clog P) < 5, hydrogen bond donor (HBD) count < 5 with hydrogen bond acceptor (HBA) count < 10, rotatable bond count (Rot B) < 10, and polar surface area (PSA) < 140*A*°^2^ [29]. Nornidulin has MW of 429.7 g/mol, Clog P 6.5, HBD 2, HBA 5, Rot B 1, and PSA 76*A*°^2^, indicating that nornidulin is an acceptable compound for development as an orally administered drug. Additionally, nornidulin showed no toxicity in T84 cells in this study, which supports the drug-like potential of nornidulin. However, the pharmacokinetic profile of nornidulin is still unknown. Therefore, further study on pharmacokinetics of nornidulin should be performed.

The mechanism of CFTR inhibition by nornidulin was investigated in this study. Our data suggest that nornidulin inhibits CFTR Cl^-^ channels activity directly. Indeed, the mechanism of direct CFTR inhibition can be explained in a comprehensive manner. Briefly, the mechanisms of well-known direct CFTR inhibitors are divided into two main modes of action: gate controlling and pore occluding mechanisms [30]. Gate controlling mode is a mechanism in which inhibitors stabilize the gate in a closed state leading to a prolonged closed time. CFTR_inh_-172 and PPQ-102 inhibit CFTR by this mechanism [31, 32]. For the pore occluding mechanism, inhibitors directly block Cl^-^ secretion by binding at the channel’s pore region, either externally or internally. GlyH-101 and MalH are using this mechanism to inhibit CFTR [15, 33]. Further studies of the direct CFTR inhibition mechanism of nornidulin should be clarified for the further development of this compound. In addition, nornidulin inhibited the FSK-induced elevation of intracellular cAMP, suggesting that mechanisms of CFTR inhibition by nornidulin may be both via a direct pathway and by lowering intracellular cAMP levels. One of the possible mechanisms is via activation of G_αi_, which function to reduce the cAMP level by inhibiting adenylate cyclases [34]. Further studies are required to test this assumption.

Several natural compounds that lower cAMP levels have been identified for treating diseases characterized by high cAMP level, such as hyperalgesia, diabetes, and polycystic kidney diseases [35–37]. These compounds act through two main mechanisms: activating phosphodiesterases (PDEs) and inhibiting adenylate cyclases (ACs). Compounds possessing PDE-activating activies include curcumin, ginsenoside, and berberine, which have been demonstrated to reduce hepatic gluconeogenesis in primary hepatocytes [38–40]. Although no natural compounds are known as AC inhibitors, ST034307, a chromone derivative, has been discovered to selectively inhibit AC1, offering potential for pain management [41, 42]. It is noteworthy that chromone, a common core structure found in various natural compounds like those from endophytic fungi and marine sponge-associated fungi, shares structural similarities with nornidulin. Exploring compounds and derivatives with chromone or depsidone-based structures could lead to identification of new AC inhibitors. However, given the crucial role of cAMP in multiple biological pathways, potential side effects must be carefully considered.

In the human colonoid model, nornidulin (10 μM) attenuated the forskolin-induced fluid secretion. However, the extent of suppression of the organoid swelling was less than that of Cl^-^ secretion in T84 cells. This may be due to the difficulty in accessibility of nornidulin to CFTR on the apical sides of intestinal epithelial cells in the colonoid model, where nornidulin was added to the basolateral sides. Furthermore, anti-secretory efficacy of nornidulin was evaluated in the colonoid models using cholera toxin (CT) as a secretagogue. We found that nornidulin, albeit at a higher concentration (40 μM), significantly suppressed the CT-induced organoid swelling. The difference in the effective concentrations of nornidulin may be resulted from different mechanisms and levels of fluid secretion induced by FSK and CT. Since cGMP is known to stimulate CFTR-mediated Cl^-^ secretion and mediate the secretory effect of heat-stable toxins (STa) from enterotoxigenic *Escherichia coli* via cGMP-protein kinase G II (PKG II)-CFTR pathway [43], we envision that nornidulin may be effective in inhibiting cGMP/STa-induced Cl^-^ or fluid secretion, which warrants further investigations.

In summary, nornidulin is identified as a novel inhibitor of CFTR Cl^-^ channels possibly by mechanisms involving direct inhibition and intracellular cAMP reduction, which is involved in the pathogenesis of secretory diarrheas. Further studies and development on nornidulin and its derivatives may provide promising sources for developing new antidiarrheal drug candidates.

## Supporting information

S1 File NMR spectroscopic data of nornidulin. (PDF)

S2 File Raw western blot. (PDF)

S3 File Minimal data set. (XLSX)

## References

[1] Organization WH. Diarrhoeal disease. 2017 May 2. Report No.

[2] Kammerer K, Higginson A, Hickey P. Recommendations for diarrhea management during medical stability operations. Mil Med. 2012;177(7):870–6. doi: 10.7205/milmed-d-12-00032 . 22808897

[3] Hirsh V, Blais N, Burkes R, Verma S, Croitoru K. Management of diarrhea induced by epidermal growth factor receptor tyrosine kinase inhibitors. Curr Oncol. 2014;21(6):329–36. doi: 10.3747/co.21.2241 . 25489260 PMC4257116

[4] Keely SJ, Barrett KE. Intestinal secretory mechanisms and diarrhea. Am J Physiol Gastrointest Liver Physiol. 2022;322(4):G405–G20. Epub 20220216. doi: 10.1152/ajpgi.00316.2021 . 35170355 PMC8917926

[5] Barrett KE, Keely SJ. Chloride secretion by the intestinal epithelium: molecular basis and regulatory aspects. Annu Rev Physiol. 2000;62:535–72. Epub 2000/06/09. doi: 10.1146/annurev.physiol.62.1.535 . 10845102

[6] Field M. Intestinal ion transport and the pathophysiology of diarrhea. J Clin Invest. 2003;111(7):931–43. Epub 2003/04/03. doi: 10.1172/JCI18326 . 12671039 PMC152597

[7] Sheppard DN, Welsh MJ. Structure and function of the CFTR chloride channel. Physiol Rev. 1999;79(1 Suppl):S23–45. doi: 10.1152/physrev.1999.79.1.S23 . 9922375

[8] Higgins CF, Linton KJ. The ATP switch model for ABC transporters. Nat Struct Mol Biol. 2004;11(10):918–26. doi: 10.1038/nsmb836 . 15452563

[9] Hegedus T, Aleksandrov A, Mengos A, Cui L, Jensen TJ, Riordan JR. Role of individual R domain phosphorylation sites in CFTR regulation by protein kinase A. Biochim Biophys Acta. 2009;1788(6):1341–9. Epub 20090326. doi: 10.1016/j.bbamem.2009.03.015 . 19328185

[10] Moon C, Zhang W, Sundaram N, Yarlagadda S, Reddy VS, Arora K, et al. Drug-induced secretory diarrhea: A role for CFTR. Pharmacol Res. 2015;102:107–12. Epub 20150930. doi: 10.1016/j.phrs.2015.08.024 . 26429773 PMC4684461

[11] Kongsuphol P, Cassidy D, Hieke B, Treharne KJ, Schreiber R, Mehta A, et al. Mechanistic insight into control of CFTR by AMPK. J Biol Chem. 2009;284(9):5645–53. doi: 10.1074/jbc.M806780200 . 19095655 PMC2645823

[12] Ren A, Zhang W, Thomas HG, Barish A, Berry S, Kiel JS, et al. A tannic acid-based medical food, Cesinex((R)), exhibits broad-spectrum antidiarrheal properties: a mechanistic and clinical study. Dig Dis Sci. 2012;57(1):99–108. Epub 20110712. doi: 10.1007/s10620-011-1821-9 . 21748285 PMC3244547

[13] Tradtrantip L, Namkung W, Verkman AS. Crofelemer, an antisecretory antidiarrheal proanthocyanidin oligomer extracted from *Croton lechleri*, targets two distinct intestinal chloride channels. Mol Pharmacol. 2010;77(1):69–78. Epub 20091006. doi: 10.1124/mol.109.061051 . 19808995 PMC2802429

[14] Yu B, Zhu X, Yang X, Jin L, Xu J, Ma T, et al. Plumbagin Prevents Secretory Diarrhea by Inhibiting CaCC and CFTR Channel Activities. Front Pharmacol. 2019;10:1181. Epub 20191009. doi: 10.3389/fphar.2019.01181 . 31649543 PMC6795057

[15] Muanprasat C, Sonawane ND, Salinas D, Taddei A, Galietta LJ, Verkman AS. Discovery of glycine hydrazide pore-occluding CFTR inhibitors: mechanism, structure-activity analysis, and in vivo efficacy. J Gen Physiol. 2004;124(2):125–37. doi: 10.1085/jgp.200409059 . 15277574 PMC2229623

[16] Pongkorpsakol P, Yimnual C, Chatsudthipong V, Rukachaisirikul V, Muanprasat C. Cellular mechanisms underlying the inhibitory effect of flufenamic acid on chloride secretion in human intestinal epithelial cells. J Pharmacol Sci. 2017;134(2):93–100. Epub 20170610. doi: 10.1016/j.jphs.2017.05.009 . 28651800

[17] Yibcharoenporn C, Chusuth P, Jakakul C, Rungrotmongkol T, Chavasiri W, Muanprasat C. Discovery of a novel chalcone derivative inhibiting CFTR chloride channel via AMPK activation and its anti-diarrheal application. J Pharmacol Sci. 2019;140(3):273–83. Epub 20190731. doi: 10.1016/j.jphs.2019.07.012 . 31444000

[18] Newman DJ, Cragg GM. Natural Products as Sources of New Drugs over the Nearly Four Decades from 01/1981 to 09/2019. J Nat Prod. 2020;83(3):770–803. Epub 20200312. doi: 10.1021/acs.jnatprod.9b01285 . 32162523

[19] Akrimajirachoote N, Satitsri S, Sommart U, Rukachaisirikul V, Muanprasat C. Inhibition of CFTR-mediated intestinal chloride secretion by a fungus-derived arthropsolide A: Mechanism of action and anti-diarrheal efficacy. Eur J Pharmacol. 2020;885:173393. Epub 20200723. doi: 10.1016/j.ejphar.2020.173393 . 32712094

[20] Muangnil P, Satitsri S, Tadpetch K, Saparpakorn P, Chatsudthipong V, Hannongbua S, et al. A fungal metabolite zearalenone as a CFTR inhibitor and potential therapy of secretory diarrheas. Biochem Pharmacol. 2018;150:293–304. Epub 20180221. doi: 10.1016/j.bcp.2018.02.024 . 29475061

[21] Noitem R, Pongkorpsakol P, Changsen C, Sukpondma Y, Tansakul C, Rukachaisirikul V, et al. Natural statin derivatives as potential therapy to reduce intestinal fluid loss in cholera. PLoS Negl Trop Dis. 2022;16(12):e0010989. Epub 20221209. doi: 10.1371/journal.pntd.0010989 . 36490300 PMC9770395

[22] Phainuphong P, Rukachaisirikul V, Phongpaichit S, Sakayaroj J, Kanjanasirirat P, Borwornpinyo S, et al. Depsides and depsidones from the soil-derived fungus *Aspergillus unguis* PSU-RSPG204. Tetrahedron. 2018;74. doi: 10.1016/j.tet.2018.07.059

[23] Foulke-Abel J, In J, Yin J, Zachos NC, Kovbasnjuk O, Estes MK, et al. Human Enteroids as a Model of Upper Small Intestinal Ion Transport Physiology and Pathophysiology. Gastroenterology. 2016;150(3):638–49 e8. Epub 20151208. doi: 10.1053/j.gastro.2015.11.047 . 26677983 PMC4766025

[24] Saetang P, Rukachaisirikul V, Phongpaichit S, Preedanon S, Sakayaroj J, Hadsadee S, et al. Antibacterial and Antifungal Polyketides from the Fungus *Aspergillus unguis* PSU-MF16. J Nat Prod. 2021;84(5):1498–506. Epub 20210416. doi: 10.1021/acs.jnatprod.0c01308 . 33861594

[25] Cahyono AW, Fitri LE, Winarsih S, Prabandari EE, Waluyo D, Pramisandi A, et al. Nornidulin, A New Inhibitor of *Plasmodium falciparum* Malate: Quinone Oxidoreductase (PfMQO) from Indonesian *Aspergillus* sp. BioMCC f.T.8501. Pharmaceuticals (Basel). 2023;16(2). Epub 20230210. doi: 10.3390/ph16020268 . 37259413 PMC9964459

[26] Dean FM, Robertson A, Roberts JC, Raper KB. Nidulin and ustin; two chlorine-containing metabolic products of *Aspergillus nidulans*. Nature. 1953;172(4372):344. doi: 10.1038/172344a0 . 13087222

[27] Blunt JW, Copp BR, Keyzers RA, Munro MH, Prinsep MR. Marine natural products. Nat Prod Rep. 2014;31(2):160–258. doi: 10.1039/c3np70117d . 24389707

[28] PubChem Compound Summary for CID 20056625, Ustin; [Internet]. National Library of Medicine (US), National Center for Biotechnology Information; 2004-. 2023 [cited 2023 Aug. 21]. https://pubchem.ncbi.nlm.nih.gov/compound/Ustin.

[29] Pollastri MP. Overview on the Rule of Five. Curr Protoc Pharmacol. 2010;Chapter 9:Unit 9 12. doi: 10.1002/0471141755.ph0912s49 . 22294375

[30] Verkman AS, Synder D, Tradtrantip L, Thiagarajah JR, Anderson MO. CFTR inhibitors. Curr Pharm Des. 2013;19(19):3529–41. doi: 10.2174/13816128113199990321 . 23331030 PMC4012685

[31] Kopeikin Z, Sohma Y, Li M, Hwang TC. On the mechanism of CFTR inhibition by a thiazolidinone derivative. J Gen Physiol. 2010;136(6):659–71. Epub 20101115. doi: 10.1085/jgp.201010518 . 21078867 PMC2995156

[32] Tradtrantip L, Sonawane ND, Namkung W, Verkman AS. Nanomolar potency pyrimido-pyrrolo-quinoxalinedione CFTR inhibitor reduces cyst size in a polycystic kidney disease model. J Med Chem. 2009;52(20):6447–55. doi: 10.1021/jm9009873 . 19785436 PMC3319430

[33] Sonawane ND, Zhao D, Zegarra-Moran O, Galietta LJ, Verkman AS. Nanomolar CFTR inhibition by pore-occluding divalent polyethylene glycol-malonic acid hydrazides. Chem Biol. 2008;15(7):718–28. doi: 10.1016/j.chembiol.2008.05.015 . 18635008 PMC3322358

[34] Dessauer CW, Chen-Goodspeed M, Chen J. Mechanism of Galpha i-mediated inhibition of type V adenylyl cyclase. J Biol Chem. 2002;277(32):28823–9. Epub 20020610. doi: 10.1074/jbc.M203962200 . 12058044

[35] Li ZH, Cui D, Qiu CJ, Song XJ. Cyclic nucleotide signaling in sensory neuron hyperexcitability and chronic pain after nerve injury. Neurobiol Pain. 2019;6:100028. Epub 20190308. doi: 10.1016/j.ynpai.2019.100028 . 31223142 PMC6565612

[36] Wang Y, Liu Q, Kang SG, Huang K, Tong T. Dietary Bioactive Ingredients Modulating the cAMP Signaling in Diabetes Treatment. Nutrients. 2021;13(9). Epub 20210830. doi: 10.3390/nu13093038 . 34578916 PMC8467569

[37] Omar F, Findlay JE, Carfray G, Allcock RW, Jiang Z, Moore C, et al. Small-molecule allosteric activators of PDE4 long form cyclic AMP phosphodiesterases. Proc Natl Acad Sci U S A. 2019;116(27):13320–9. Epub 20190617. doi: 10.1073/pnas.1822113116 . 31209056 PMC6613170

[38] Zhang B, Pan Y, Xu L, Tang D, Dorfman RG, Zhou Q, et al. Berberine promotes glucose uptake and inhibits gluconeogenesis by inhibiting deacetylase SIRT3. Endocrine. 2018;62(3):576–87. Epub 20180816. doi: 10.1007/s12020-018-1689-y . 30117113

[39] Xiao N, Lou MD, Lu YT, Yang LL, Liu Q, Liu B, et al. Ginsenoside Rg5 attenuates hepatic glucagon response via suppression of succinate-associated HIF-1alpha induction in HFD-fed mice. Diabetologia. 2017;60(6):1084–93. Epub 20170309. doi: 10.1007/s00125-017-4238-y . 28280902

[40] Wang Z, Xu D, She L, Zhang Y, Wei Q, Aa J, et al. Curcumin restrains hepatic glucose production by blocking cAMP/PKA signaling and reducing acetyl CoA accumulation in high-fat diet (HFD)-fed mice. Mol Cell Endocrinol. 2018;474:127–36. Epub 20180227. doi: 10.1016/j.mce.2018.02.018 . 29499209

[41] Sengupta S, Mehta G. Natural products as modulators of the cyclic-AMP pathway: evaluation and synthesis of lead compounds. Org Biomol Chem. 2018;16(35):6372–90. doi: 10.1039/c8ob01388h . 30140804

[42] Brust TF, Alongkronrusmee D, Soto-Velasquez M, Baldwin TA, Ye Z, Dai M, et al. Identification of a selective small-molecule inhibitor of type 1 adenylyl cyclase activity with analgesic properties. Sci Signal. 2017;10(467). Epub 20170221. doi: 10.1126/scisignal.aah5381 . 28223412 PMC5734633

[43] Wang H, Zhong Z, Luo Y, Cox E, Devriendt B. Heat-Stable Enterotoxins of Enterotoxigenic *Escherichia coli* and Their Impact on Host Immunity. Toxins (Basel). 2019;11(1). Epub 20190108. doi: 10.3390/toxins11010024 . 30626031 PMC6356903

