# Supplementary material for: Inhibition of CFTR-mediated intestinal chloride secretion by nornidulin: Cellular mechanisms and anti-secretory efficacy in human intestinal epithelial cells and human colonoids
Source: PLoS One. 2024 Dec 23;19(12):e0314723. doi: 10.1371/journal.pone.0314723 (PMC11665983; doi:10.1371/journal.pone.0314723)
Supplement: S1 File — (PDF) [file pone.0314723.s001.pdf]

Supporting Information File 1

NMR spectroscopic data of nornidulin

^1H and ^{13}C NMR spectra of nornidulin were recorded on a 300 MHz Bruker FTNMR Ultra Shield spectrometer using standard Bruker pulse sequences with a concentration of 1% w/v in $CDCl_3$. Chemical shifts are expressed in δ (parts per million, ppm) referring to the tetramethylsilane peak.

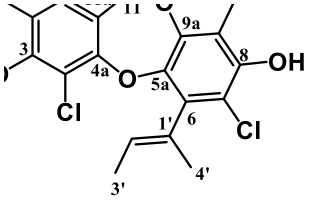

Table The ^1H and ^{13}C NMR spectroscopic data of nornidulin in $CDCl_3$

Position	δ_H (*mult.*, J_{Hz})	δ_C (C-Type)	Position	δ_H (*mult.*, J_{Hz})	δ_C (C-Type)
1		140.1 (C)	8-OH	5.81 (*brs*)	
1-Me	2.51 (*s*)	18.8 (CH_3)	9		115.5 (C)
2		115.9 (C)	9-Me	2.30 (*s*)	10.0 (CH_3)
3		151.8 (C)	9a		142.4 (C)
4		110.1 (C)	11		161.8 (C)
4a		157.4 (C)	11a		119.5 (C)
5a		142.0 (C)	1′		129.3 (C)
6		134.6 (C)	2′	5.44 (*qq*, 6.9, 1.5)	128.3 (CH)
7		115.9 (C)	3′	1.82 (*dq*, 6.9, 1.2)	14.1 (CH_3)
8		147.9 (C)	4′	1.96 (*quint*, 1.2)	17.4 (CH_3)

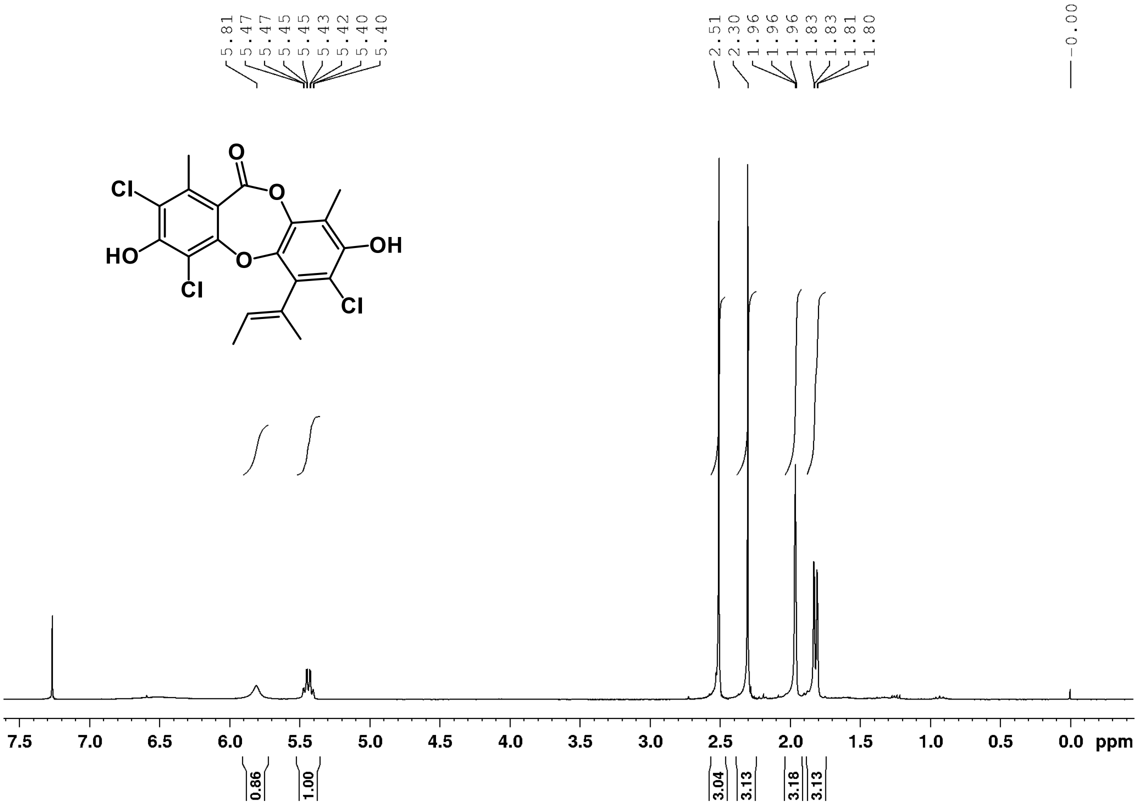

The ¹H NMR (300 MHz) ($CDCl_3$) spectrum of nornidulin

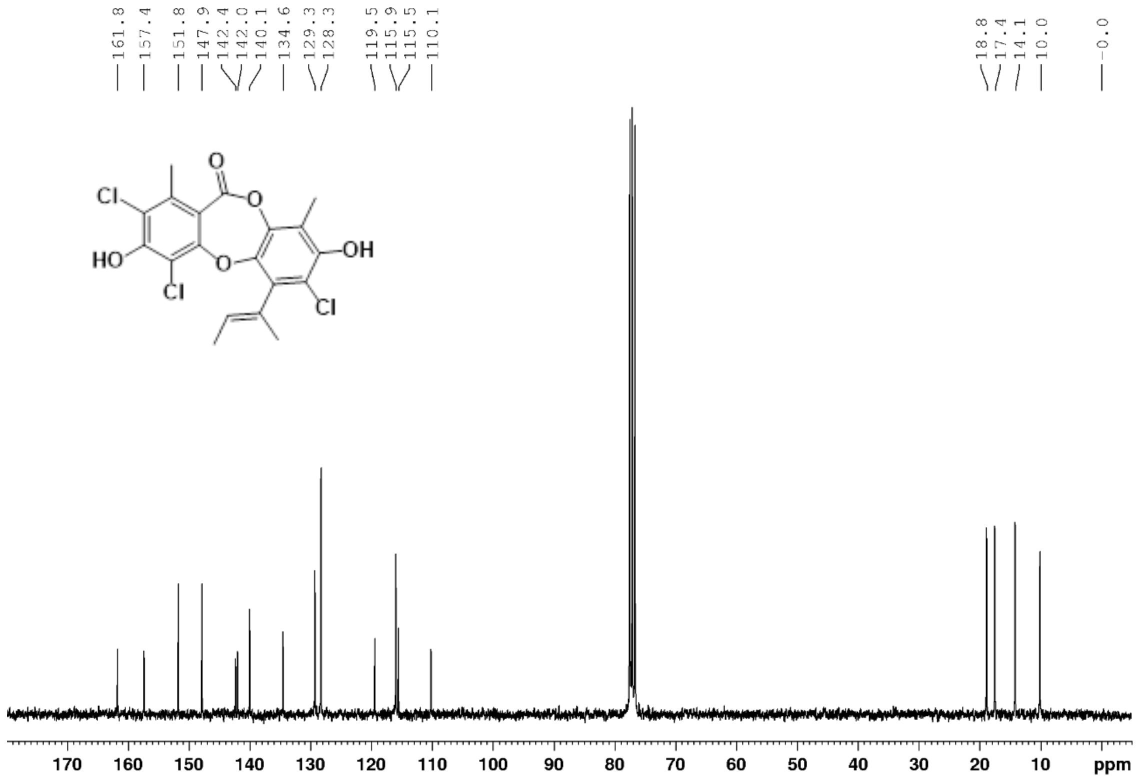

The ¹³C NMR (75 MHz) ($CDCl_3$) spectrum of nornidulin
