# Supplementary material for: Inhibition of CFTR-mediated intestinal chloride secretion by nornidulin: Cellular mechanisms and anti-secretory efficacy in human intestinal epithelial cells and human colonoids
Source: PLoS One. 2024 Dec 23;19(12):e0314723. doi: 10.1371/journal.pone.0314723 (PMC11665983; doi:10.1371/journal.pone.0314723)

CFTR : 168 kDa

N1 : Control , Nornidulin / N2 : Control , Nornidulin / N3 : Control , Nornidulin

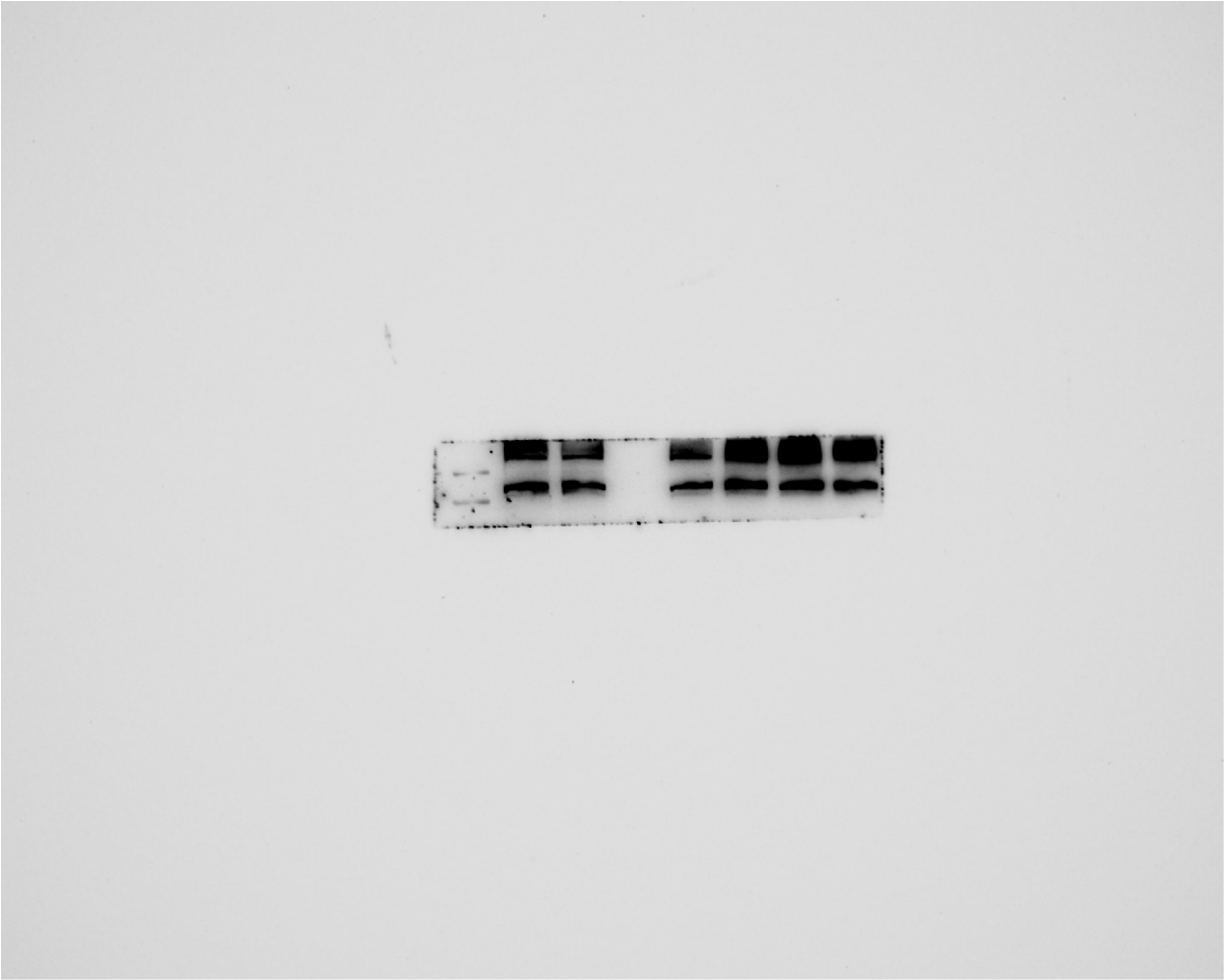

Beta - Actin : 45 kDa

N1 : Control , Nornidulin / N2 : Control , Nornidulin / N3 : Control , Nornidulin

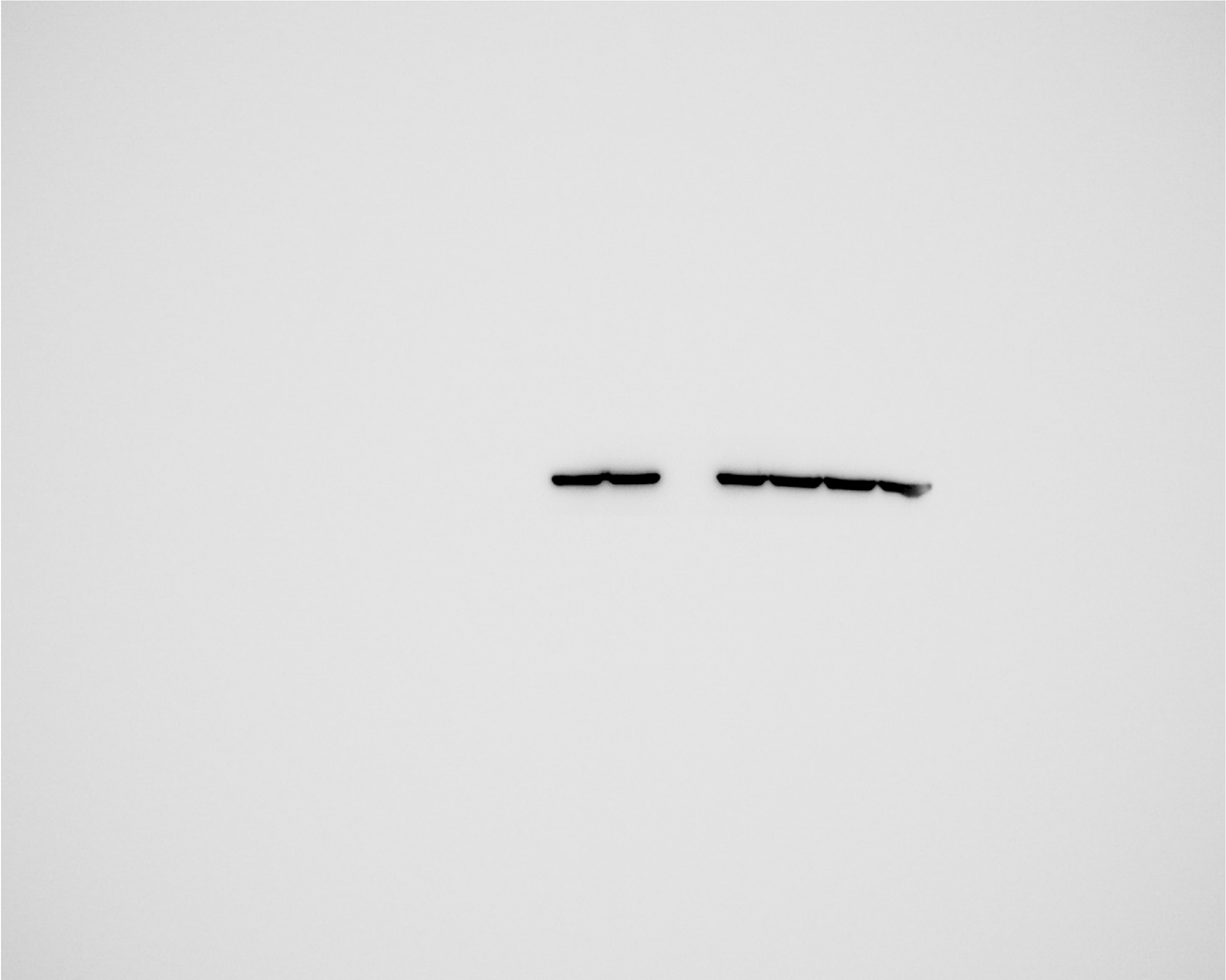

CFTR : 168 kDa

N4 : Control , Nornidulin

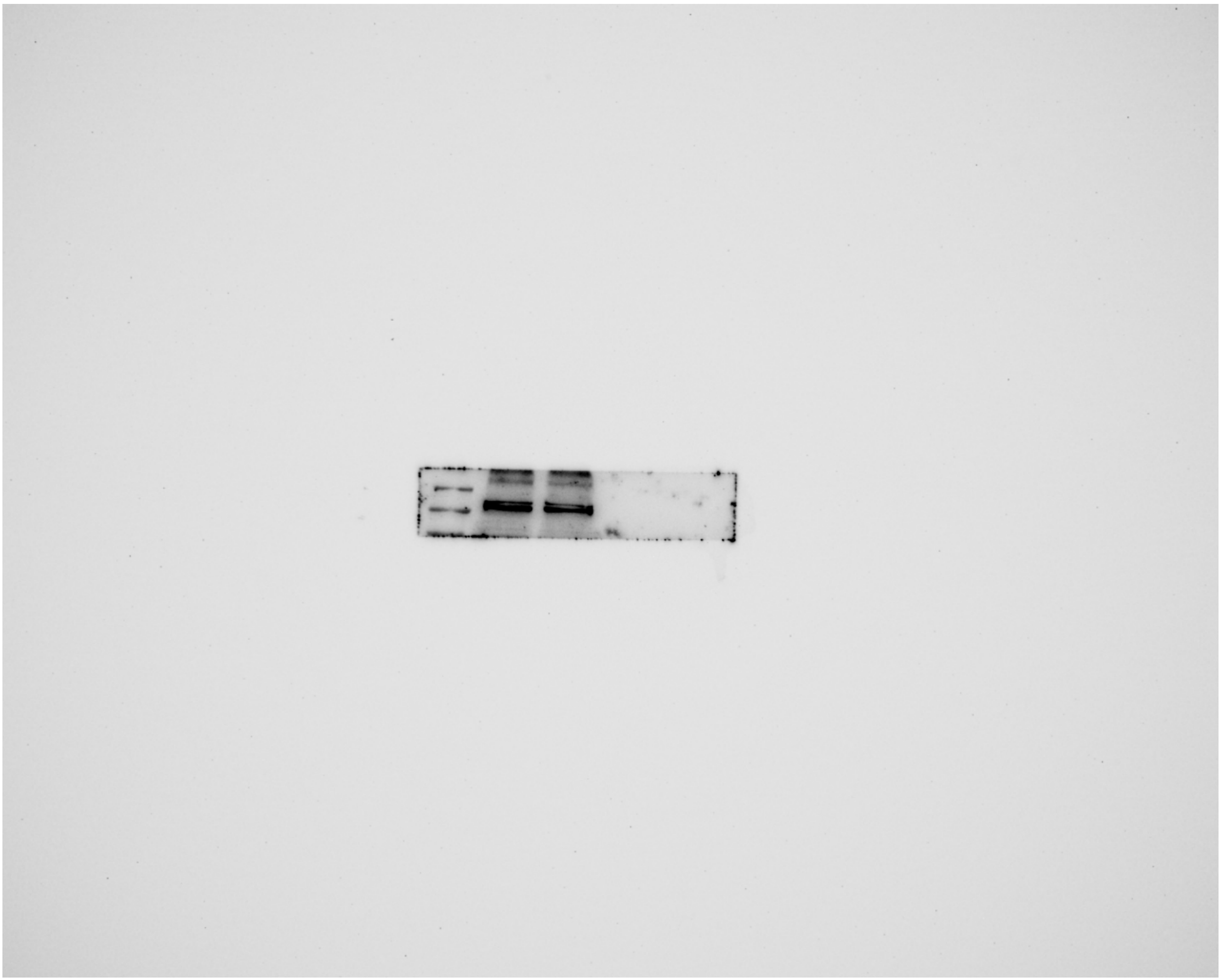

Beta - Actin : 45 kDa

N4 : Control , Nornidulin

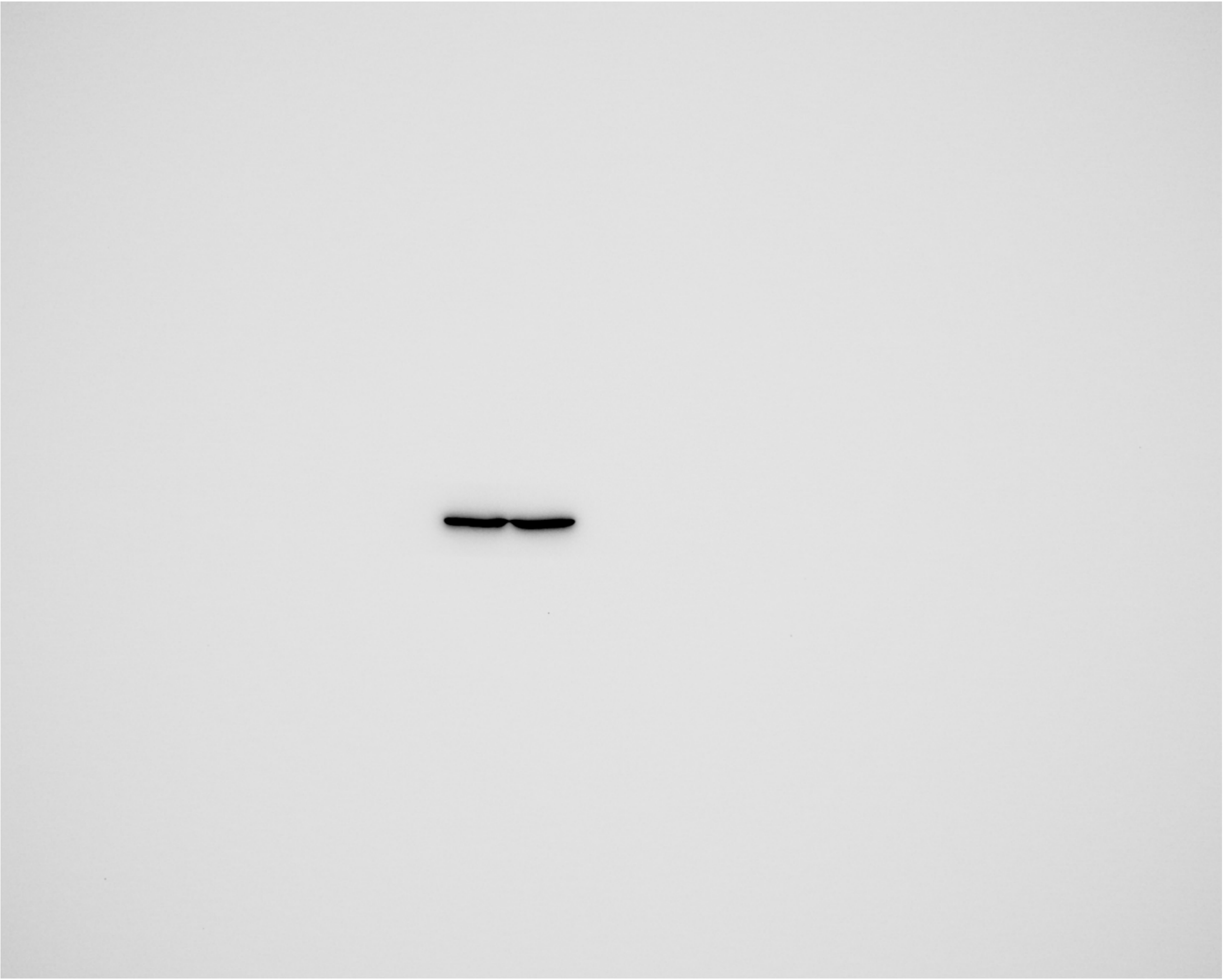

CFTR : 168 kDa

N5 : Control , Nornidulin

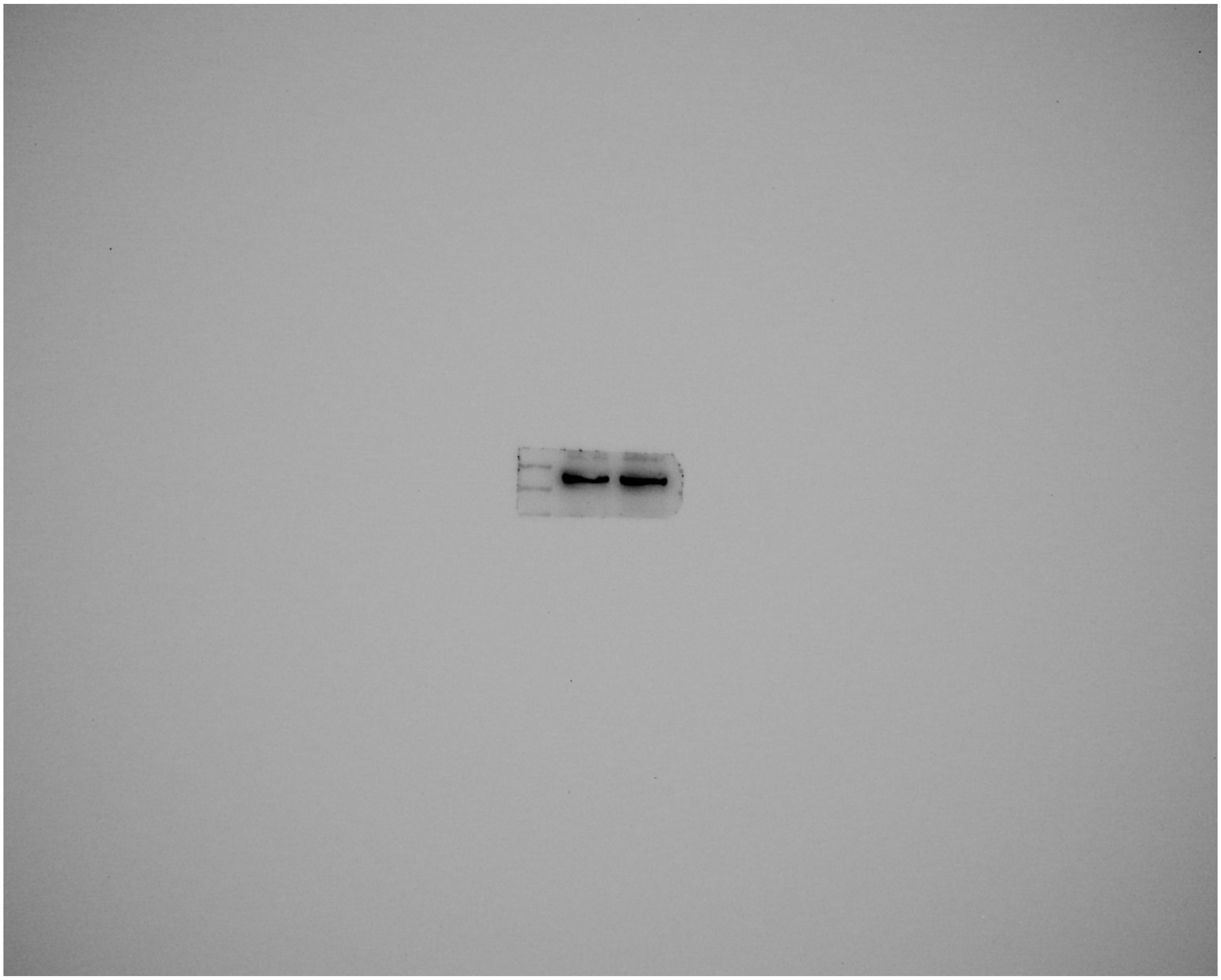

Actin : 45 kDa

N5 : Control , Nornidulin

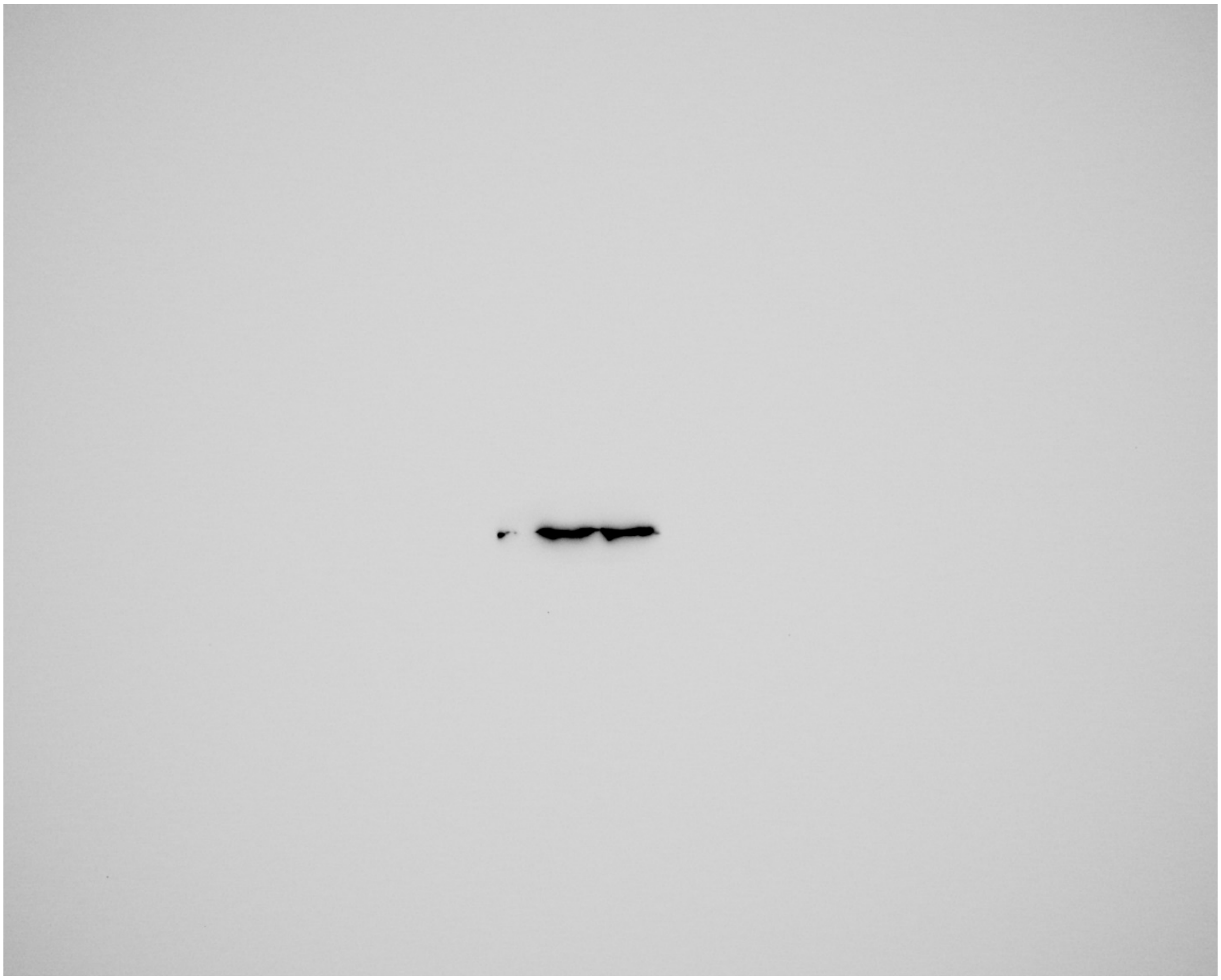

CFTR : 168 kDa

N6 : Control , Nornidulin

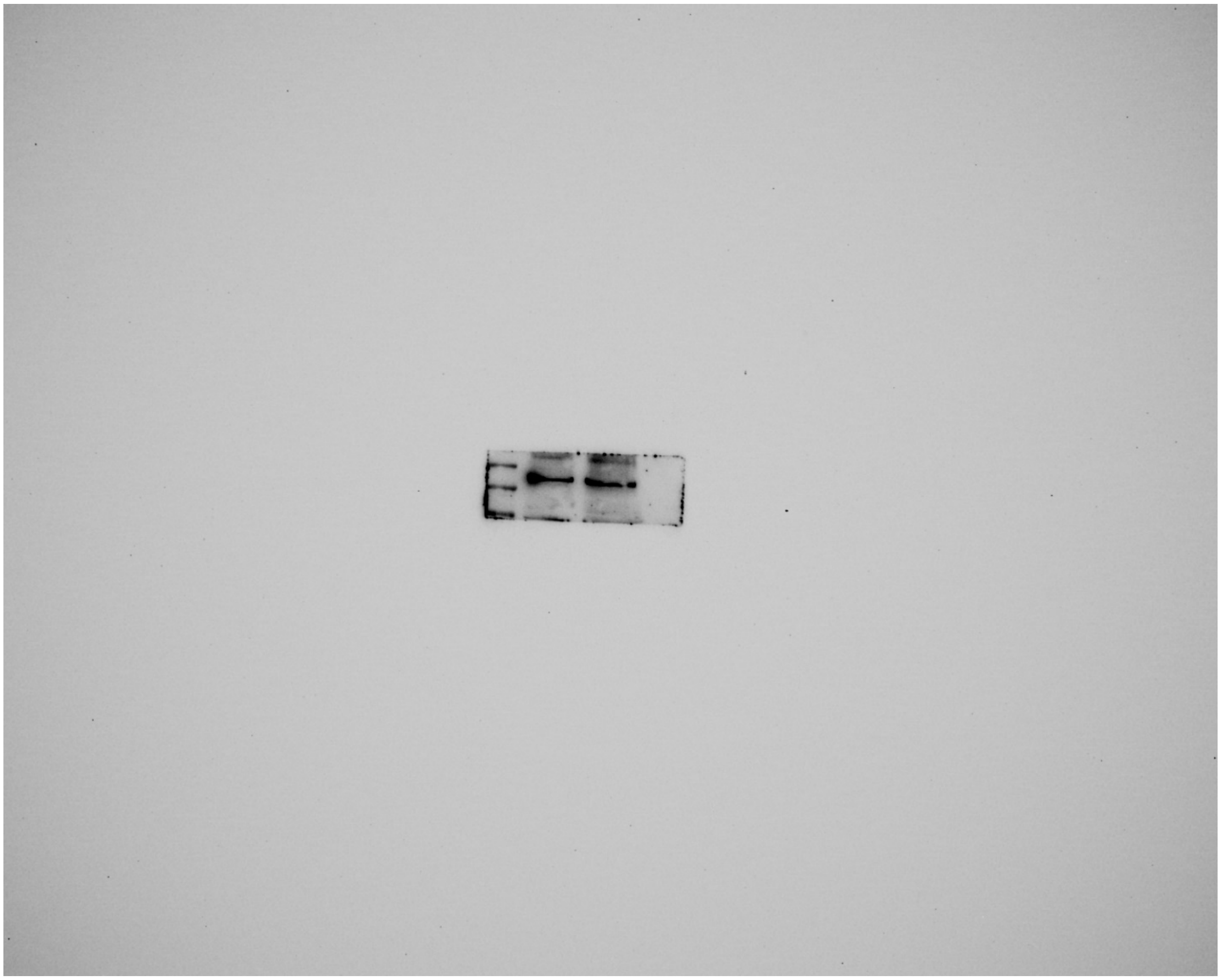

Actin :45 kDa

N6 : Control , Nornidulin

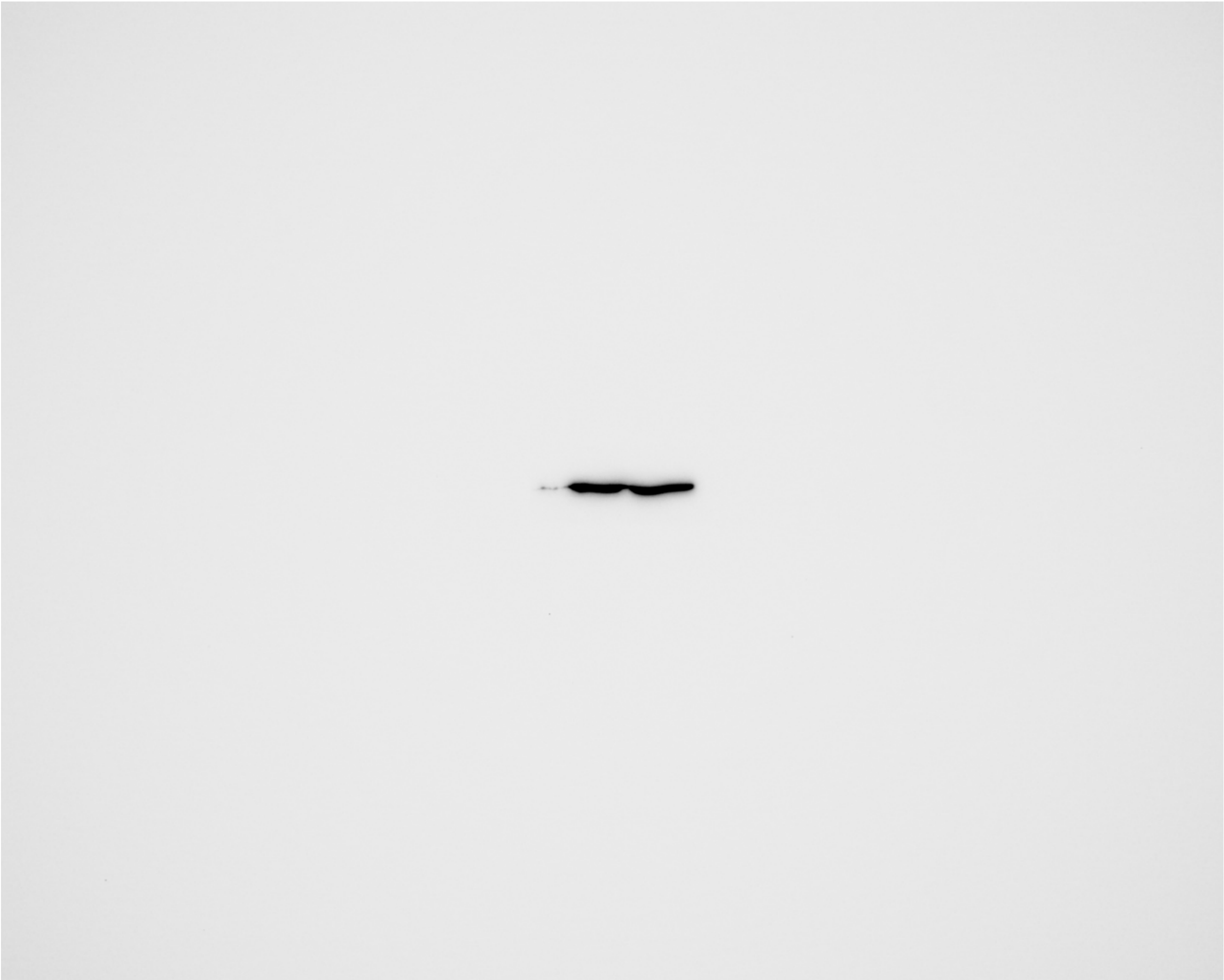

CFTR : 168 kDa

N7 : Control , Nornidulin

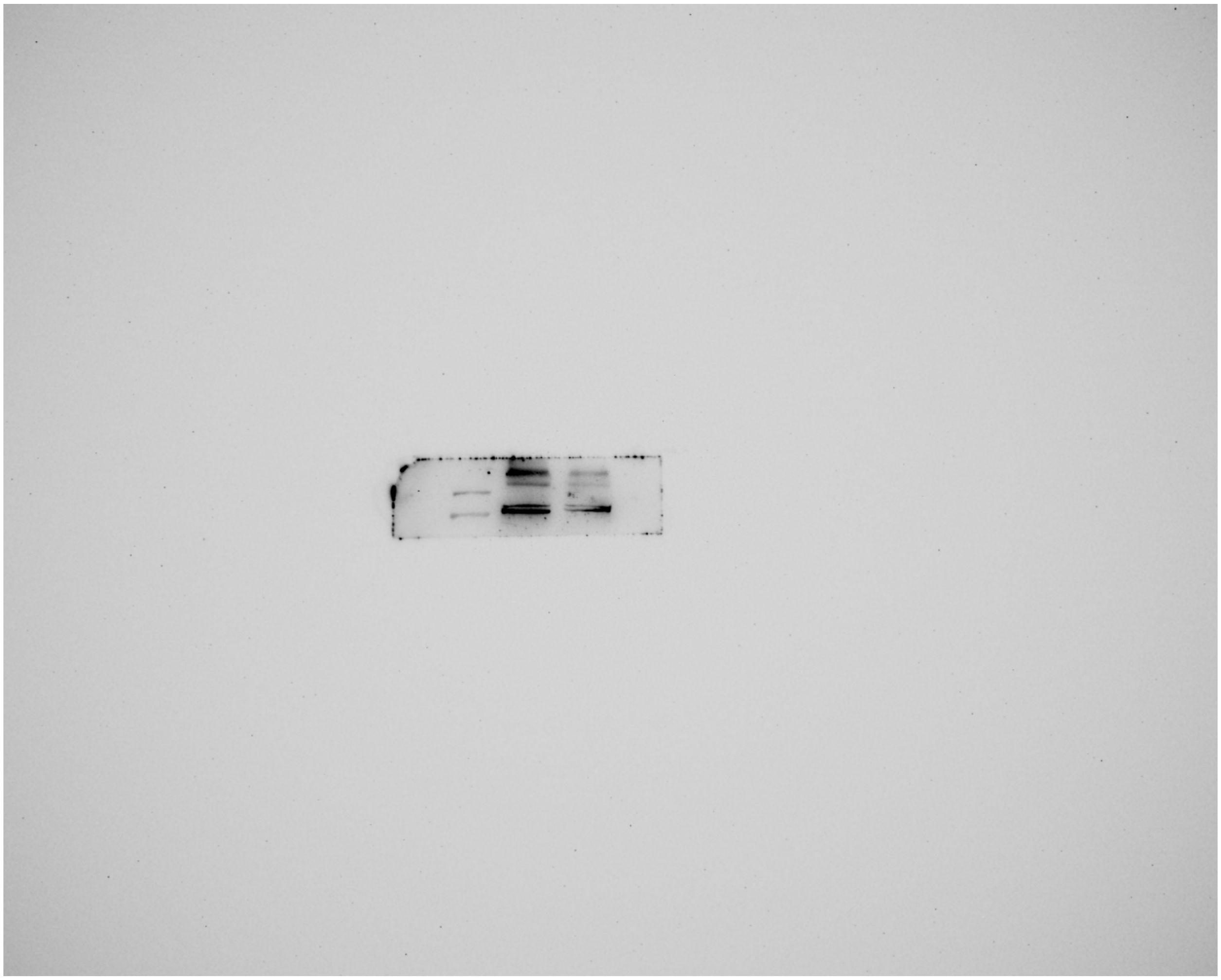

Actin :45 kDa

N7 : Control , Nornidulin

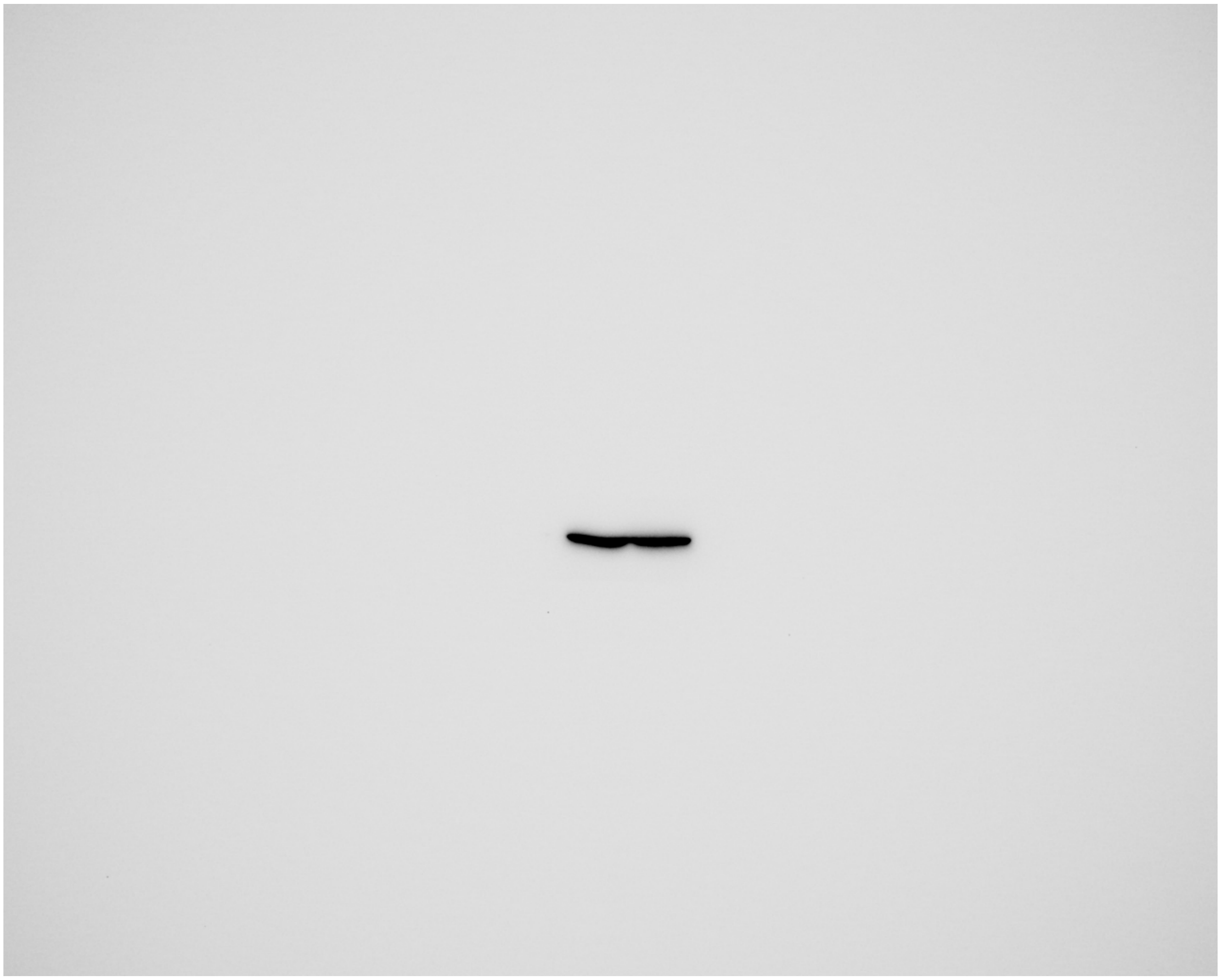

CFTR : 168 kDa

N8 : Control , Nornidulin

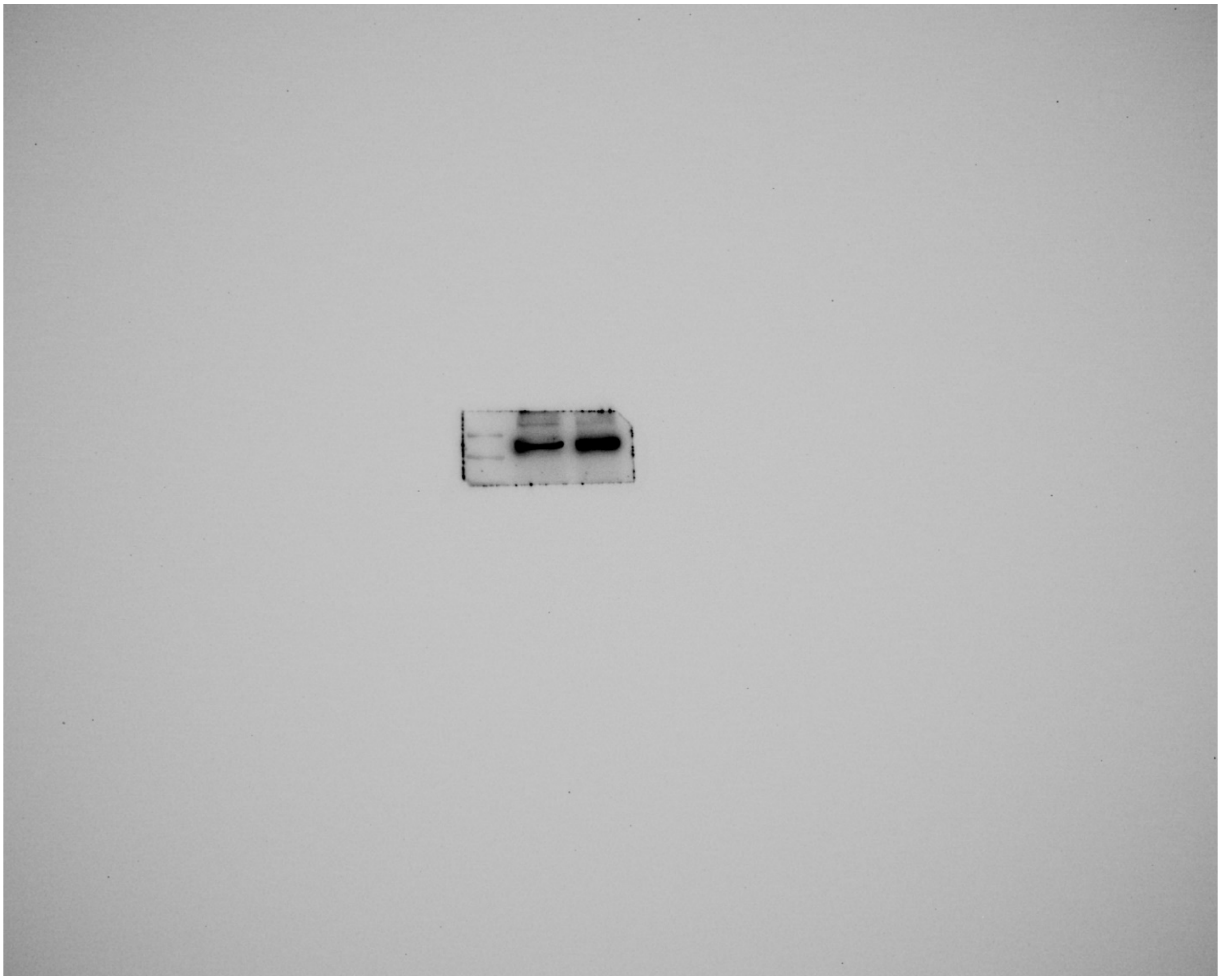

Actin :45 kDa

N8 : Control , Nornidulin

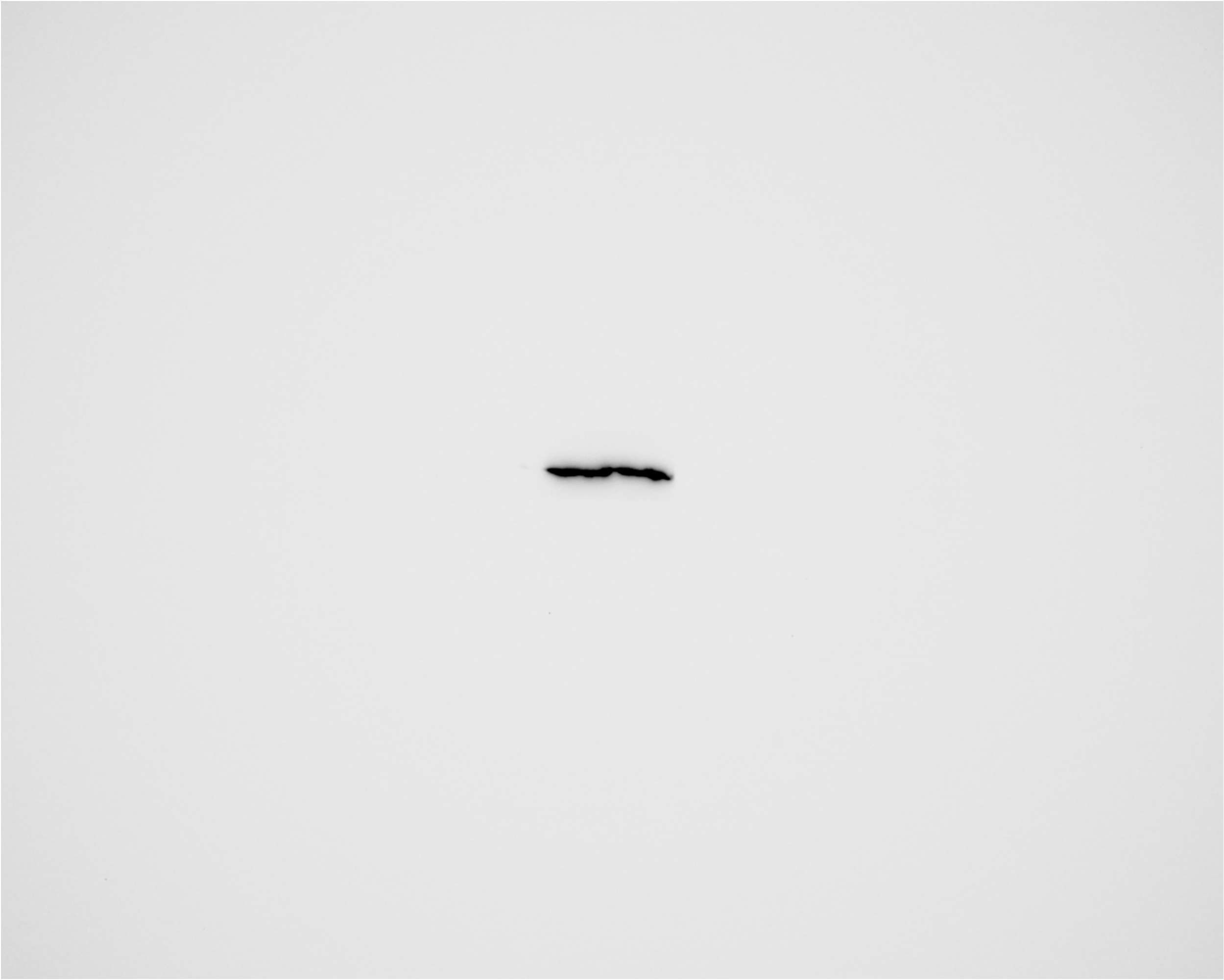

Supplement: S2 File — (PDF) [file pone.0314723.s002.pdf]
